# Resilience and social change: Findings from research trends using association rule mining

**DOI:** 10.1016/j.heliyon.2023.e18766

**Published:** 2023-07-27

**Authors:** Cheongil Kim, Jaesun Yeom, Seunghoo Jeong, Ji-Bum Chung

**Affiliations:** a School of Urban and Environmental Engineering, Ulsan National Institute of Science and Technology (UNIST), Ulsan, Republic of Korea; b School of Business Administration, Ulsan National Institute of Science and Technology (UNIST), Ulsan, Republic of Korea; c Advanced Railroad Civil Engineering Division, Korea Railroad Research Institute (KRRI), Uiwang, 16105, Republic of Korea

**Keywords:** Resilience, Resilience thinking, Social change, Research trends, Association rule mining, Bibliometric analysis

## Abstract

This study analyzed the historical development of *resilience* with respect to multidisciplinary aspects using association rule mining (ARM). ARM is a rule-based machine-learning approach tailored to identify validated relations among multiple variables in a large dataset. This study collected author keywords from all *resilience*-related literature in the Web of Science database and examined the changes in validated *resilience*-related topics using ARM. We found that *resilience*-related research tends to diversify and expand over time. Although topics and their academic fields related to engineering and complex adaptive systems were prominent in the early 2000s, *psychosocial resilience* and *social-ecological resilience* have received significant attention in recent years. The increasing interest in *resilience*-related topics linked to psychological and ecological factors, as well as social system components, can be attributed to the impact of a series of complex and global events that occurred in the late 2000s. Recently, *resilience* has been conceived as a way of thinking, perspective, or paradigm to address emergent complexity and uncertainty with vague concepts. *Resilience* is increasingly being regarded as a boundary spanner that promotes communication and collaboration among stakeholders who share different interests and scientific knowledge.

## 1 Introduction

The concept of *resilience* has become complex and diversified across multiple disciplines, even within the same research field [[1], [2], [3]]. The meaning of *resilience* contains vague [4], metaphoric [5], and malleable [1] concept, as its usage has been expanded. Such conceptual vagueness has facilitated a rapid spread of this concept to a variety of research fields [[1], [2], [3],[6], [7], [8], [9], [10], [11], [12], [13], [14], [15], [16]]. Since the 2000s, *resilience thinking* has been popularized as an alternative anticipation-based strategy against global risks, including climate change, terrorism, and infectious disease [17,18]. The term *resilience* has been adopted as a universal norm based on *resilience thinking* to realize sustainability under threats to human societies [19,20].

Literature reviews have provided comprehensive insights into *resilience* in various academic areas, such as climate change [8,10], disaster management [[21], [22], [23]], energy policy [24], business [9], health care services [25], and community systems [13]. Currently, there is a significant interest in *resilience* amidst the COVID-19 pandemic, as it plays a crucial role in addressing various socioecological issues, such as mental health [26], healthcare services [27], international trade [28], and food security [29]. Extensive *resilience*-related review studies have addressed the following issues by subjectively interpreting selected papers: definition and conceptual framework [9,[11], [12], [13]], *resilience*-related indicators [11,12,22,23], role of *resilience* [[30], [31], [32]], and assessment models for quantifying the level of resilience [11,22,23,32]. Additionally, some reviews have employed bibliometric analysis to systematically review research trends in resilience [8,10,21,24]. Network analysis is commonly utilized in bibliometric analysis to visualize relationship structures among targets of interest, such as topics [33,34]. Previous bibliometric reviews have analyzed the quantitative and relational aspects of *resilience*-related studies covering number of publications [8,24], spotlighted topics [8,10,21,24,25], influential authors or countries [25,35], and citation levels [8,24,25,35].

We identified several research gaps that need to be considered for an in-depth understanding of the *concept of resilience* as universal thinking. First, few frameworks exist for understanding *resilience* as a shared concept across multidisciplinary fields. Most prior studies have focused on a specific research area to gain knowledge of *resilience* despite its broad adoption in various disciplines [[8], [9], [10],^13^,^21^,^24^,^25^]. Second, it is difficult to capture the historical development of *resilience* as universal terminology in terms of its utilization and expansion in various disciplines over time. As previous studies have provided knowledge on *resilience* within specific fields of interest, it may be challenging to adopt it in emerging issues of multidisciplinary aspects owing to the lack of frameworks for historical evolution. Third, it has been challenging to identify changes in the co-studied research topics in *resilience*-related studies that show a research trend. Previous bibliometric studies have examined quantitative changes in spotlighted topics, but have methodological limitations in exploring validated topic pairs co-studied over time. Furthermore, the names of the academic disciplines must be unified. A specific field may be denoted differently because few systematic criteria may not be apparent to acquire knowledge in this field [36]. Therefore, further study is required to advance the current boundaries of *resilience*-related knowledge by expanding this concept into universal thinking.

This study aims to understand the historical development of *resilience* in the context of multidisciplinary aspects. As scholars have interpreted *resilience* differently, this study focused on tracking spotlighted co-studied topics and corresponding research fields. We collected author keywords from all *resilience*-related scientific publications and examined changes in *resilience*-related topics using association rule mining (ARM). ARM is a widely used machine learning method that extracts statistically validated item pairs. By examining the association rules from the author keywords, validated topic pairs closely related to the concept of *resilience* can be identified. The results of this study can provide scientific knowledge on the dominant co-studied topics and corresponding academic fields in *resilience*-related studies by time and disciplinary boundaries, and expansion of *resilience thinking* over time by associating with specific worldwide events and social change.

## 2 Research background

### 2.1 Expansion of resilience thinking

While the meaning of *resilience* has a long history, the term itself was initially utilized in academic contexts during the 1970s [37]. The origins of the *resilience* concept have remained a subject of controversy among scholars; however, it started to gain significant prominence particularly within the field of ecology [38]. Most scholars attribute the introduction of *resilience* to ecologist C. S. Holling in 1973 [38]. During that time, the term of *resilience* emerged as a way to describe the ability of individuals, materials, or systems to recover and return to their original state following a negative shock or disturbance [[37], [38], [39]].

The idea of *resilience* has expanded to the social domain since the second half of the 20th century. As our society has faced global changes in terms of globalization, urbanization, and climate change, risk factors related to social, environmental, and technical aspects have become severe and complex. It has been challenging not only to pinpoint the causal relationship of damage incurred by risks but also to manage the uncertainty of future risks [19]. R*esilience* has received significant attention as a means of enhancing one's ability to manage complex risks.

Successive catastrophes, such as the 2005 Hurricane Katrina, the 2008 World Financial Crisis, the 2011 East Japan Earthquake and COVID-19 pandemic [19] have facilitated the adoption of *resilience thinking*, which focuses on understanding the interrelationship between humans and the environment based on a systematic approach [40,41]. *Resilience thinking* emerged as a response to the limitations of traditional system dynamics in ecology [40,42,43], which disregarded the reality of multiple stable states and ongoing changes [44,45]. Recognizing the interaction between social and ecological systems, the term of *resilience* has been increasingly viewed within the framework of a complex adaptive system (CAS) [46,47].

The expansion of *resilience thinking* can be explained by the following reasons. First, the existing anticipation-oriented risk management showed limitations in addressing potential risks which becomes complex and uncertain [48]. Insufficient anticipation capabilities and the unpredictable nature of global changes present formidable obstacles in accurately forecasting the magnitude and likelihood of future risks, consequently impeding the development of effective risk mitigation strategies [48,49]. Second, the term of *resilience* has been broadly adopted as a universal norm to realize sustainability under the substantial impact of global risks on human societies [[17], [18], [19]]. In recent years, risk strategies have increasingly emphasized *resilience* as a means to enhance the capacity of systems to absorb, adapt, and recover through learning and reconstruction [19,20]. Third, globalization, its connection with informatization, has fostered the adoption of *resilience* to address changes in socio-ecological systems (SES). Globalization, characterized by rising connectivity, increased speed, spatial stretching, and declining diversity, has posed global challenges such as epidemiological threats, invasive species, financial collapses, and global terrorist networks [41]. To address such unpredictable changes, there has been increased interest in developing a framework to improve *resilience* [40,50] through learning and self-organization against change and chaos [51].

### 2.2 Resilience as boundary object and bridging concept

*Resilience* with vague [4], metaphoric [5], and malleable [1] concepts has been referred to as either *boundary object* or *bridging concept*, reflecting how scholars understand it in their fields [1]. The term of *resilience* as a *boundary object* or *bridging concept* is interpreted in a broader meaning across disciplines as a ‘way of thinking’ [43]. It is conceived as a perspective or even a paradigm for analyzing complex systems rather than as a well-defined concept [43,52,53].

An entity may be the *boundary object* when it is interpreted with various meanings shared among diverse communities that possess unique vocabularies [54] due to conceptual vagueness [1,3]. The meaning of *resilience* under the *boundary object* is likely to be of interpretative flexibility over time [55]. Interpretative flexibility can facilitate communication across various fields and collaboration among multiple groups by sharing the same vocabulary with different meanings [1,[55], [56], [57]].

The proponents of the *bridging concept* argue that the *resilience* term serves as a catalyst, facilitating the linkage between science and policy [3,58] as well as promoting communication across diverse scientific fields and systems [3,58,59]. With its ability to bridge disciplinary boundaries, *resilience thinking* in natural science has the potential to be transferred to the social context and effectively utilized in planning and policymaking [58].

### 2.3 Limitations of previous reviews

This study observed several research gaps that require attention, drawing insights from previous review studies across diverse academic fields. To pinpoint these gaps, we examined selected publications by authors that conducted comprehensive reviews of *resilience*-related research within their respective domains, which is summarized in Table 1.

**Table 1:** Examples of *resilience*-related review studies.

Field	Purpose	Method	Implication
Climate change [8,10]	• Explore the production of knowledge in the field of climate change	• Bibliometric analysis (proportions of publications, co-citation patterns, keyword links)	• Guide future research on climate change integration with other fields • Develop shared understanding of *resilience* under disaster risk reduction and climate change adaptation philosophy
Disaster [8,13,21]	• Explore trends in scientific outputs and contributions regarding the *resilience* agenda	• Systematic literature review on the historical development of disaster *resilience* • Bibliometric analysis (keywords, citation and co-citation, institutions, country-wise analyses)	• Identify hotspots in diverse disaster *resilience* research • Bridge concept between fields • Guide development, implementation, and evaluation of disaster and community *resilience* policies
Urban [12,13,60]	• Create a conceptual framework to support the production of urban *resilience* tools	• Systematic literature review on the scientific and technical literature about urban *resilience* • Bibliometric analysis (co-citation network)	• Systematic approach to urban *resilience* for building cities • Foster *resilience* in urban settings and encourage collaboration among researchers and stakeholders • Act as a boundary object for defining urban resilience
Energy [11,24]	• Clarify the concept of energy *resilience* and propose a taxonomy	• Systematic literature review on characters, indicators, and formula of energy system *resilience* • Bibliometric analysis (core author, productive counties, keywords, co-citation)	• Trace conceptual contributions and identify gaps in energy system *resilience* research • Provide opportunities for future research on energy resilience frameworks and modeling methods
Organization [9]	• Analyze the definition of *resilience* and elaborate a novel conceptual framework on *resilience* of firms	• Systematic literature review on *resilience* in business and management field	• Contribute to *resilience* literature on firms with absorptive and adaptive resilience paths
Safety [31]	• Examine how the peer-reviewed safety science constructs *resilience* as a scientific object	• Systematic literature review on *resilience* in organization and safety	• Highlight complexities of socio-technical systems and the need for *resilience* engineering studies • Raise ethical questions for safety science field
Industrial ecology [35]	• Review *resilience* and complexity in industrial ecology and the broader academy	• Systematic literature review on *resilience* definitions excluding medical and health science • Bibliometric analysis (co-citation network of the literature on *resilience and complexity*, and industrial ecology and *resilience*	• Promote dynamic *resilience* principles in industrial ecology • Facilitate interdisciplinary collaboration and advance *resilience* scholarship
Socio-ecology [7,61]	• Analyze the *resilience*-related research trends of ecological, economic, social, and integrated socioecological systems	• Bibliometric analysis (number of publications per year, rank of journals, co-citation, co-authors, top productive countries, and distribution of case areas covered in *resilience* publications)	• Broaden application of *resilience* to socioeconomic systems and sustainability science • Drive urgent action-oriented solutions for ecological degradation
Business [62]	• Analyze research trends in the supply chain *resilience*	• Bibliometric analysis (keywords, co-authors, co-citation network) • Systematic literature review on supply chain resilience in SMEs in the context of the coronavirus (COVID-19) pandemic	• Provide foundation for future supply chain *resilience* studies • Expand research scope in various subfields of supply chain *resilience*
Psychology [30]	• Synthesize studies on *resilience*, coping behaviors and social support among health care workers during the COVID-19 pandemic	• Systematic literature review on selected papers regarding “psychological *resilience*”, “mental health”, “healthcare workers”, “social support”, and “COVID- 19″	• Inform nursing management and strategies for enhancing *resilience* and social support in healthcare workers' mental health
Health care [25]	• Identify how resilient health care (RHC) literature developed over time	• Bibliometric analysis (keywords, co-authors, and definition references network) • Systematic literature review on concepts, stages, analytical frameworks, and implementation of health system *resilience*	• Understand growth and changes in resilient health care field • Shift focus towards generating *resilience* in healthcare organizations • Provide refinements to the current understanding of health system *resilience* considering the COVID-19 pandemic

First, it is difficult to identify *resilience*-related research trends across multidisciplinary fields because previous studies have focused on domain-centric reviews. As shown in Table 1, *resilience* has been scrutinized within a single research field. Each review focused on analyzing the emergence of *resilience* and research trends within a specific field. Therefore, previous studies have been limited to providing scientific evidence that *resilience* can be used as a shared concept (i.e., *boundary object* or *bridging concept*) in various fields. Amid a paradigm shift in which *resilience thinking* has been expanding, further research is required to identify the *resilience*-related ideas that have been shared across various research fields.

Second, few studies have examined the purpose of *resilience* adoption and its expansion, which makes it difficult to extract the risks or uncertainties that require *resilience* as an idea. According to Table 1, bibliometric analysis is a widely used method for examining quantitative phenomena (e.g., the number of publications) and relational attributes (e.g., networks of keywords and authors) of *resilience*-related studies. Previous studies have helped researchers and policymakers move forward to a more resilient society by providing bibliometric research trends. Although these studies contributed to alleviating risks by suggesting the concept of *resilience* to major issues in each field, they were limited to enhancing the scientific basis and justification for expanding the *resilience* approach and *resilience thinking* in various fields.

Third, previous bibliometric studies have encountered methodological challenges when investigating the dynamics of co-studied *resilience*-related topics. While network analysis has been employed in previous studies to capture changes in spotlighted research topics over time, it has fallen short in elucidating the changes in research topics that were co-studied alongside a specific topic. For a deeper understanding of the trajectory of a research topic, it is crucial to comprehend the changes in associated research topics that have validated connections. Furthermore, previous methods are often constrained by predefined research question, which can limit exploratory research focus [63]. The identified limitations underscore the need for a more comprehensive approach that transcends quantitative analysis.

In addition, the name of the academic field is not systematic but subjective; that is, it can be expressed differently expressed by authors, even representing the same academic discipline. For example, *socio-ecology* is denoted as *a social-ecological system* [64] or *a social and ecology* [7]. Different expressions with the same terminology may be confusing to obtain *resilience*-related ideas as a *boundary object* or a *bridging concept* [36].

## 3 Research framework

**Fig. 1:**
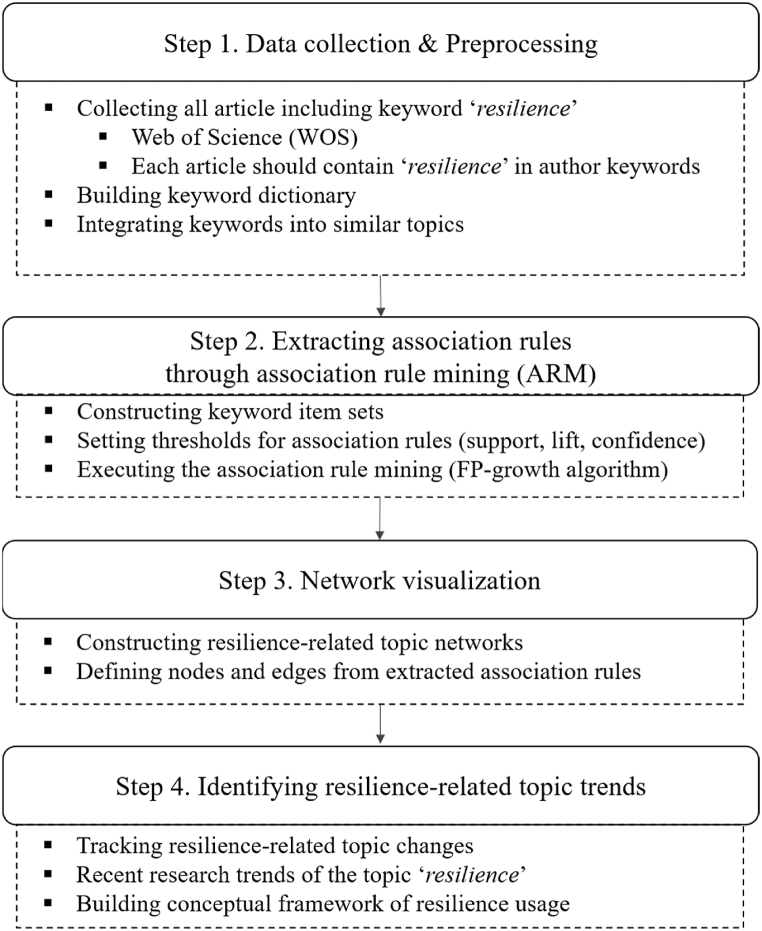
Research framework for *resilience*-related trend analysis.

We propose a framework for identifying *resilience*-related research trends by adopting ARM and network analysis (Fig. 1). The proposed framework comprises four steps as follows.

Step 1: Data collection & preprocessing.

To identify *resilience* research trends, *resilience*-related articles were collected from the Web of Science (WOS) database. The WOS is the Clarivate Analytics web database that contains comprehensive citation data for various academic disciplines. The database is a well-known bibliometric data source that covers 79 million core collections and 171 million platforms, and provides bibliometric data including title, author keywords, abstracts, publication years, and other metadata of research papers.

R*esilience*-related research publications were collected using the advanced search function provided by the WOS webpage. As the goal of this study is to examine historical development of *resilience* in the context of multidisciplinary aspects, the articles should include the ‘*resilience’* keyword in author keywords. Specifically, the current study collected 14,843 research papers from 2001 to 2020 and gradually filtered out unrelated articles. After excluding book chapters and proceedings, 12,959 articles were remained in our final dataset. Specifically, this study extracted bibliometric information such as title, authors, keywords, publication year, and WOS categories. The publication years ranged from 2001 to 2020. The reason for setting 20 years as the target period window is threefold. First, the time window should be sufficient to indicate the changes in *resilience*-related research trends in an obvious manner. A 20-year window would provide enough information about the changes in *resilience* concepts and usage patterns. Second, drastic social changes in response to climate change and COVID-19 have occurred over the last 10 years. As these events have drawn attention and influenced society, it is worthwhile to understand recent changes in the concept of *resilience* by tracing 20-year trends. Third, the number of publications before 2001 was insufficient for bibliometric analysis (206 from 1990 to 2000). As the concept of *resilience* has become multidisciplinary in recent years, there is little advantage in extending the time window beyond 20 years.

After collecting bibliometric information, we constructed a topic dictionary to eliminate the linguistic ambiguity. Linguistic ambiguity is twofold: (1) semantic ambiguity and (2) morphological ambiguity. With linguistic ambiguities, research trends cannot be accurately identified creating bias in quantifying topic keywords. Semantic ambiguity arises from the multiple representations of similar concepts. For example, the keywords ‘depressive symptoms’ and ‘depressive disorder’ can be categorized using the concept of ‘depression’. These keywords have a common core concept but occur in different linguistic formats. Such linguistic ambiguity can be eliminated by categorizing these keywords into a single representative subject. Morphological ambiguities are caused by various grammatical formations. For instance, the keywords ‘coronavirus’ and ‘COVID-19’ are both synonyms represented in different ways. In addition, the keywords ‘decision making’ and ‘decision-making’ indicate identical subjects, but the dash mark between the two consecutive words raises morphological ambiguity. Such ambiguities can be eliminated by integrating these keywords into a single topic.

Constructing a topic dictionary can minimize bias in keyword quantification. A semi-automated method was employed to create a keyword dictionary. This method automatically clusters similar keywords based on their inclusion in a stem corpus. After clustering the keywords, representative topics were allocated to each keyword cluster according to the consensus of the authors. The topic dictionary consists of 522 topics with 773 unique keywords.

Step 2: Extracting association rules through ARM.

This step extracts statistically validated rules from *resilience* research topics by adopting the ARM, an efficient technique for constructing edge-centric networks. The ARM is a rule-based machine learning approach for identifying statistically strong linkages among multiple variables in a large dataset [65]. An association rule indicates how often an event occurs and how closely variables are related. The algorithm searches for frequent item sets by evaluating and pruning the item subsets. To execute ARM and evaluate the extracted rules, three probabilistic indicators were used: support*, confidence*, and *lift*. Using these measures, statistically significant association rules (co-occurrence of *resilience* topics) can be identified.

To conduct ARM, this study built the topic distribution of each paper based on the topic dictionary from the previous step. Each author keyword was converted into a unique integrated topic from the topic dictionary so that each paper could have tangible research keywords. Because the articles cover *resilience*-related subjects from various perspectives, the set of tangible keywords provides a reference for research fields on *resilience*-related topics.

This study utilized the Python package *mlxtend* [66], which includes the functions required to conduct ARM. The function *fpgrowth* creates a frequent pattern tree from the preprocessed topic basket extracted from the previous step, which is essential for identifying validated patterns among *resilience*-related topics. To facilitate the calculation process, we filtered out association rules that appeared less than three times. If topics *A* and *B* are only included in two papers; the association rule between *A* and *B* is less likely to be considered in the research field.

Step 3: Network visualizations.

This step constructs topic networks based on the ARM rules. As the association rules consist of keywords that represent validated co-occurrence topics, the nodes of the network were the keywords, and the edges indicated their connection as a rule. For example, if the extracted association rules were (1) *recovery → resilience* and (2) *trauma → resilience*, three nodes (*recovery*, *resilience*, and *trauma*) and two edges were generated. Topic networks from multiple timeframes provide graphical information for tracing the trends of *resilience*-related studies.

Topic networks for four timeframes (i.e., 2005, 2010, 2015, and 2020) were presented to visualize the relationships between rule-based topics. We selected a five-year interval period based on the worldwide occurrences of big events, such as global agreements, that is, the Sustainable Development Goals, the Paris Agreement for Climate Change, the Sendai Framework for Disaster Risk Reduction (2015), and the COVID-19 pandemic (2020), to track the global trends in *resilience*-related studies. Based on the expansion of *resilience thinking*, the selected periods can represent the timing of global events that facilitate *resilience*-related studies. When drawing the networks, this study set the support value as the weight of the edges in the networks. To visualize outstanding topics, the edges with less than 0.01 of support as their weights were eliminated.

Step 4: Identifying resilience-related research trends.

This step identifies *resilience*-related topic trends by exploring antecedent topic*s* linked to consequent topic *resilience*. As the networks provide validated *resilience* research topics graphically, extracting *resilience* as a consequent topic in networks is essential for understanding the changes in rule-based topics co-occurring with *resilience*. The dominant topics in each year were determined by sorting degrees of support in descending order. If the same support value was observed among various topics, then the study achieved a higher rank on a topic with a higher *confidence* level. Rules with the same support and *confidence* levels were regarded as having the same ranks. Tracing changes in rule-based topics provides information about trends in *resilience*-related studies.

As each scientific publication belongs to a specific WOS research category, it is possible to identify significant research categories in *resilience*-related studies by using ARM-based rules. The WOS category comprised 252 segmented subject areas in science, social science, arts, and humanities. This study extracted the spotlighted research categories by counting the frequency of each category appearing in publications with validated rules. Both rule-based topics and their corresponding research categories provide scientific knowledge on the historical development of *resilience* in various research disciplines.

## 4 Results

This section presents ARM-based multidisciplinary research trends in *resilience* in terms of spotlighted topics and their corresponding research categories. First, we analyzed quantitative changes in *resilience*-related studies regarding publication numbers, validated rules, and research categories over 20 years. Topic networks were constructed to illustrate the relationship between the ARM-based rules. Second, this study investigated yearly variations in spotlighted topics that were co-studied with *resilience*. Third, research categories containing ARM-based topics were introduced to extract the dominant research fields that focused on *resilience*. The results of this study can clarify the historical development of *resilience*-related studies over the past 20 years, providing scientific evidence for the expansion of *resilience thinking*.

### 4.1 Overall research trends

**Fig. 2:**
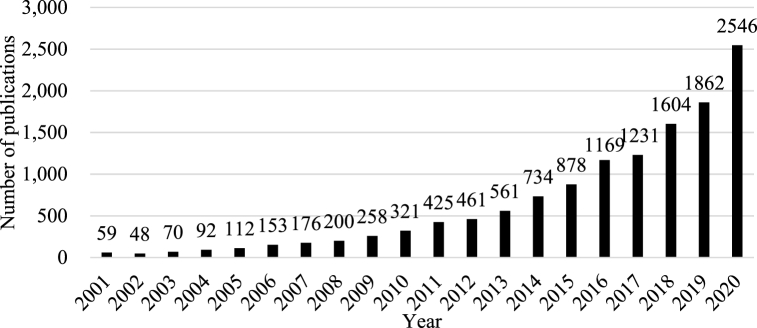
Number of *resilience*-related publications by year.

The annual changes in the number of publications between 2001 and 2020 are shown in Fig. 2. A total of 12,960 scientific papers on *resilience* has been published over the last 20 years. The number of publications gradually increased in mid-2010. After 2015, the number showed an exponential growth pattern and reached 2546 in 2020, more than twice the number in 2016. This increasing trend is attributable to the expansion of *resilience thinking* caused by globalization, a more complex world, and frequent extreme events.

**Fig. 3:**
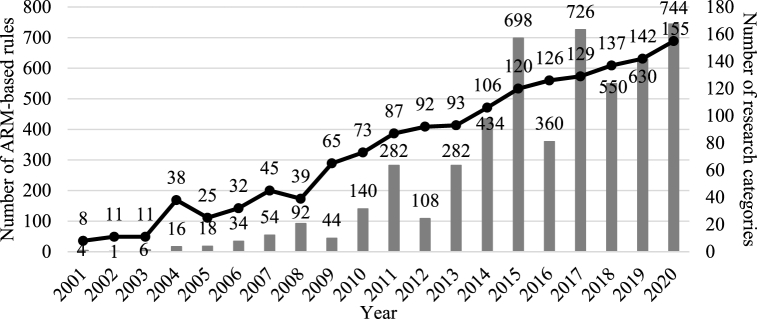
Number of validated rules (bar) and corresponding research categories (line).

Both the number of validated topic rules and research categories have increased since 2001 (Fig. 3). ARM-based topic rules with a support of over 0.002 were selected to clarify the readability of the graph. After identifying the validated rules, we counted the number of unique research categories containing each rule. The number of rules increased continuously, except in 2004 and 2008, reaching a peak value of 744 in 2020. Although up-and-down patterns were observed, the number of research categories showed an overall increasing trend similar to that of the number of rules. Of the 252 research categories in the WOS, 191 (76%) were unique fields related to *resilience*-related issues over 20 years.

**Fig. 4:**
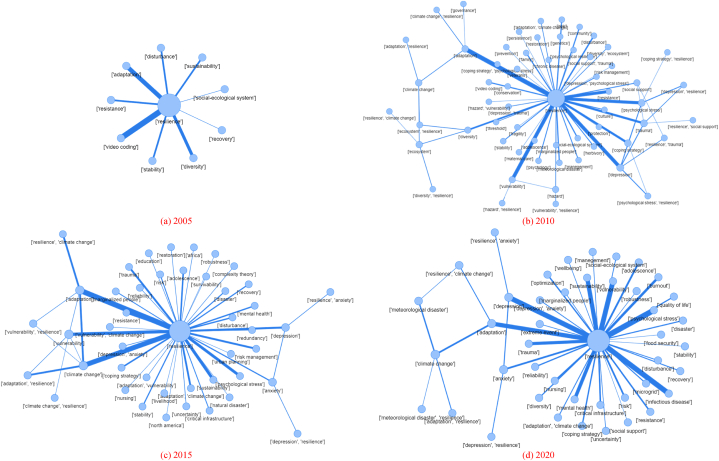
Association rule mining (ARM)-based validated research topics - network representation.

This study constructed topic networks for four years (2005, 2010, 2015, and 2020) to identify the relationship between validated rules (Fig. 4). The thicker the edge in the network, the more two topic pairs co-appeared in the journal articles. The topic network for 2005 was sufficiently simple to identify validated topic rules based on edge thickness. The network size in other time frames (i.e., 2010, 2015, and 2020) was larger than that in 2005, indicating an increase in research interest in *resilience*-related studies in various research fields. The topic network can provide graphical evidence that *resilience* has received increasing interest over time; however, there are challenges in identifying statistically significant topic rules when the network is complicated.

### 4.2 ARM-based research topic trends

We presented representative *resilience*-related topics by selecting rules containing *resilience* as the consequent topic with a support higher than 0.01. The topic rules with the top 10 ranks are summarized in Table 2. The yearly extracted antecedent topics along with the ARM indices are listed in Appendix A, and the yearly variations in topic rankings are indicated in Appendix B. The yearly rankings of the research categories are presented in Appendix C.

**Table 2:** Top 10 antecedent topics co-studied with *resilience* in 2005, 2010, 2015, and 2020.

Rank	2005	2010	2015	2020
1	- video coding	- adaptation	- adaptation	- psychological stress
2	- adaptation	- vulnerability	- climate change	- infectious disease
3	- diversity - sustainability	- depression	- vulnerability	- depression
4	- disturbance	- resistance	- sustainability	- vulnerability
5	- stability - resistance	- psychological stress	- psychological stress	- sustainability
6	- social-ecological system - recovery	- coping strategy	- depression	- adaptation
7		- trauma	- resistance	- mental health
8		- diversity	- trauma	- trauma
9		- disturbance	- risk management	- burnout
10		- adolescence	- adaptation, climate change	- adolescence

In 2005, socio-ecological research topics such as *adaptation*, *diversity*, *stability*, and *social-ecological system* had high rankings. Although *video coding* was the dominant topic related to *resilience*, its rank declined to 12th position in 2010 and disappeared in 2015 and 2020. *Video coding* has been widely utilized to deal with communication-related issues such as videos, intranets, and mobile satellite systems. After five years, *adaptation*, which had the 2nd rank in 2005, became the most validated topic. *Vulnerability* was the 2nd most frequently mentioned topic and remained one of the important topics in 2015 and 2020 with 3rd and 4th ranks, respectively. In 2015, *adaptation* and *climate change* became the 1st and 2nd significant topics, respectively, indicating an increased research interest in climate change-related studies. In 2020, *psychological stress*, *infectious disease*, and *depression* ranked high, confirming the effects of the COVID-19 pandemic. The ARM-based results provide meaningful evidence that rule-based research topics change over time based on research interest in each period.

**Fig. 5:**
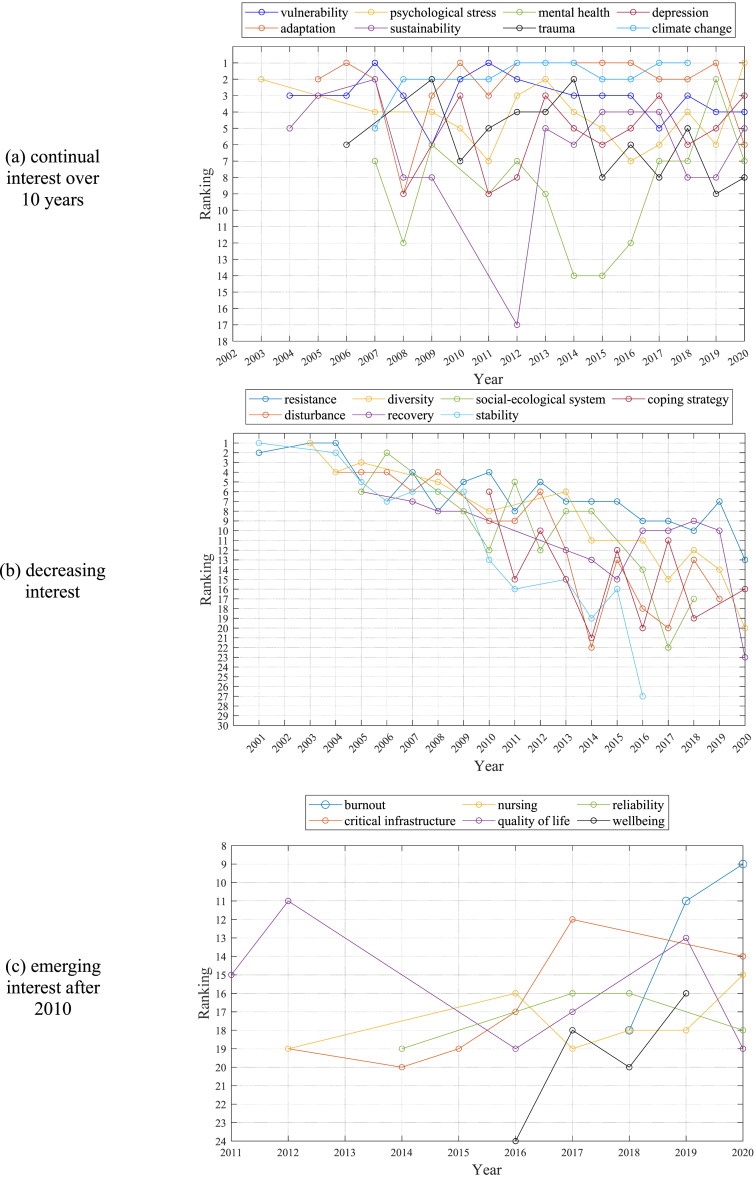
Association rule mining (ARM)-based research topic trends from 2001 to 2020.

Rule-based topic trends can be classified into three patterns based on their level of interest: continual, decreasing, and emerging. This study analyzed attention received by a single topic in *resilience*-related publications over the past 20 years (Fig. 5). Topics that have received attention over 10 years include *vulnerability*, *psychological stress*, *mental health*, *depression*, *adaptation*, *sustainability*, *trauma*, and *climate change*. Topics with continual interest were likely to have relatively fewer fluctuating rankings compared with other rule-based topics.

A decreasing interest can be observed in several topics, such as *resistance*, *diversity*, *social-ecological system*, *coping strategy*, *disturbance*, *recovery*, and *stability.* S*tability* was the most spotlighted topic in 2001, while its interest decreased to 27th rank in 2016 and fell out of ranking after 2016. Research interest in *resistance* declined from the 2nd rank in 2001 to the 13th rank in 2020 at a relatively slower rate compared with other topics.

While there were topics with decreasing focus, those with emerging interest in recent years have also been observed. New research topics such as *burnout*, *nursing*, *reliability*, *critical infrastructure*, *quality of life*, and *wellbeing* have begun to appear in rule-based topic lists. *Burnout* began to receive focus in 2018 with the 18th ranking and became the 9th most important topic in 2020. The dramatic increase in interest in *burnout* in 2020 may have been affected by the spread of the COVID-19 pandemic worldwide.

### 4.3 ARM-based research category trends

**Fig. 6:**
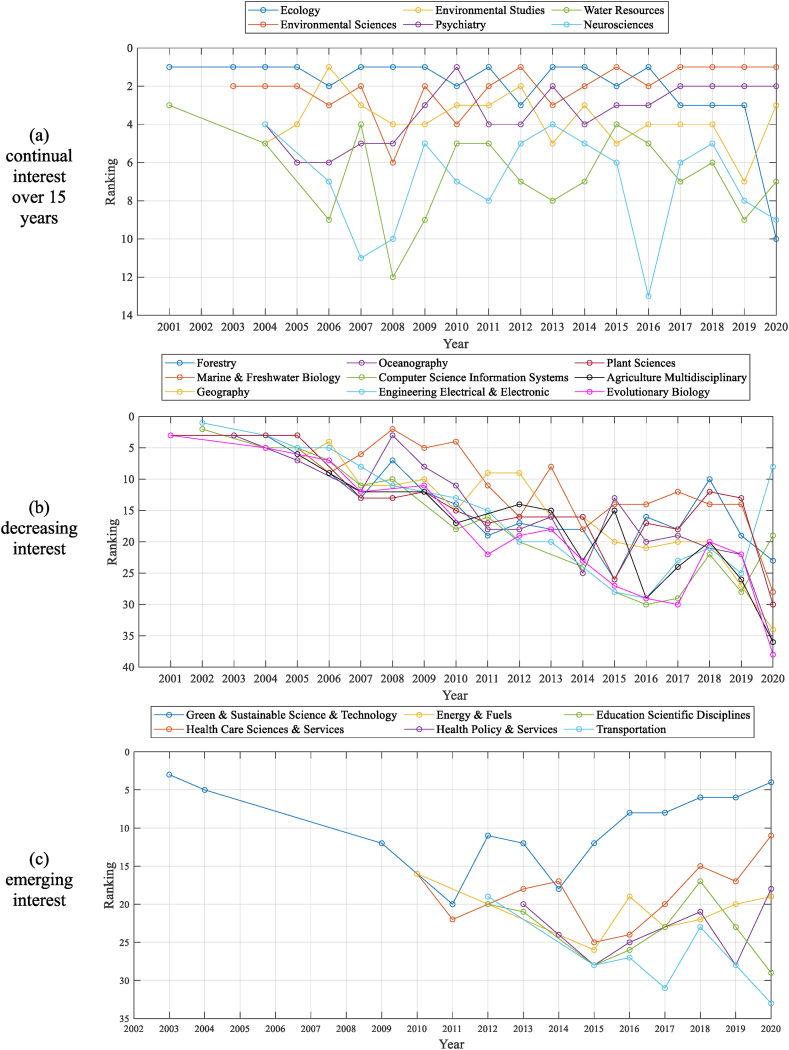
Association rule mining (ARM)-based research category trends from 2001 to 2020. Although the types of research categories have diversified, as explained in Fig. 3, several categories, such as *Forestry*, *Oceanography*, *Plant Sciences*, and *Marine & Freshwater Biology*, have received decreasing interest (Fig. 6b). Categories such as *Energy & Fuels*, *Education Scientific Disciplines*, *Health Care Sciences & Services, Health Policy & Services,* and *Transportation* have emerged as significant *resilience*-related societies since 2010 (Fig. 6c). Interest in *Green & Sustainable Science & Technology* was low prior to 2010 but has been ranked as an important category every year since 2011. As ARM-based research topics are linked to the corresponding research categories, we can understand in-depth *resilience*-related multidisciplinary research trends in terms of both topics and research categories over 20 years based on bibliographic data.

The ARM-based research categories can be grouped into three trends: continual, decreasing, and emerging interests (Fig. 6). Examples of research categories of continual interest include *Ecology*, *Environmental Studies*, *Water Resources*, *Environmental Sciences*, *Psychiatry*, and *Neurosciences*. *Ecology* was one of the significant categories, ranging from first to third rank, except in 2020. *Environmental Sciences* has been the most dominant category in *resilience*-related publications holding first rank in 2017, followed by *Psychiatry* in 2nd position. Research categories with decreasing interests were also observed.

## 5 Discussion

### 5.1 Diversification of resilience-related topics

*Resilience*-related topics diversified throughout the research period. At the beginning of the 21st century, informatization became a new paradigm, enabling the world to become an information society through the adoption of information and communication technologies (ICTs) [67]. ICTs such as virtual compositions (e.g., multimedia) and mobile communication was highlighted as a research interest, which can be observed by the topic of *video coding* in the early 2000s. Under the effect of informatization, representative topics in 2005 were related to *engineering resilience* (e.g., *video coding*, *recovery*, *disturbance*, *stability*, and *resistance*). However, topics related to *psychosocial resilience* and *social-ecological resilience* stood out as spotlighted interests in 2010 (e.g., *adaptation*, *vulnerability*, *depression*, *psychological stress*, *trauma*), which can be attributed to the impact of a series of global-scale events, such as Hurricane Katrina in 2005, the World Financial Crisis in 2008, and the East Japan Earthquake in 2011. Furthermore, topics such as *risk management*, *infectious diseases*, *mental health*, *burnout*, *critical infrastructure*, and *quality of life* emerged in 2015 and 2020 (see Appendices A). Topics related to climate change adaptation, mitigation, and maintenance of diversity (e.g., *adaptation*, *climate change*, *vulnerability*, *disturbance*, *and resistance*) received significant attention in the mid-2010s, while topics related to psychological well-being and health (e.g., *psychological stress*, *depression*, *trauma*, *mental health*, *anxiety*, *burnout*, *and infectious disease*) were likely to have more interest in the late 2010s. The emergence of *infectious diseases* as a new *resilience*-related topic in 2020 may be attributed to the worldwide spread of COVID-19, first reported at the end of 2019.

The findings of this study showed that the concept of *resilience* has evolved into two main discourses: *social-ecological* and *psycho-social resilience*. These discourses have received a significant interest due to growing uncertainty associated with social and environmental changes [68]. Despite little consensus on its definition or theory, we can conclude that *resilience* concept has played a critical role in bridging disciplines, fostering collaboration, and offering potential for further advancements. According to Fig. 5a and b, *resilience*-related research has focused on topics within *social-ecological* and *psycho-social resilience* discourses (e.g., *vulnerability*, *psychological stress*, *mental health*, *depression*, *adaptation*, *sustainability*, *and trauma*). *Resilience thinking*, related to issues of psychological well-being and healthy living, has become more important in recent years. Increasing interest in *burnout*, *nursing*, *reliability*, *critical infrastructure*, *quality of life*, *wellbeing* since 2010 was shown in Fig. 5c. Given the growing complexity and uncertainty, the *resilience* approach has been increasingly adopted as a tool to achieve sustainable development [17,18] by focusing on the interaction between the environment and human-built systems [69].

### 5.2 Multidisciplinary research fields

As specific *resilience*-related topics are increasingly being discussed in various research fields, *resilience* has become a perspective for analyzing complex systems rather than a clear and well-defined concept [1,53]. R*esilience* acts as a boundary spanner connecting broader research fields and topics.

As shown in Fig. 6a, there has been a steady interest in *resilience* in fields related to the environment and mental health over 15 years (e.g., *Ecology*, *Environmental Studies*, *Water Resources*, *Environmental Sciences*, *Psychiatry*, *Neurosciences*). This indicates that *resilience* has been studied in a wide range of fields within two broad parallel discourses: *social-ecological resilience* and *psychosocial resilience*. Since 2010, multidisciplinary research has been conducted to link social sciences with ecological and psychological variables. For example, this study found that *adaptation* has been mainly studied in *Ecology*, *Environmental Sciences*, and *Environmental Studies*, and *depression* has been mentioned in *Psychiatry*, *Clinical Neurology*, *Neurosciences*, and *Public Environmental & Occupational Health*.

### 5.3 Expansion of resilience thinking

The number of ARM-based research fields increased from 2001 to 2020, showing clear evidence for the expansion of *resilience thinking*. In addition, we empirically proved that *resilience thinking* has expanded to various fields since the 2000s based on ARM-based topics and research fields. There are two main reasons for this finding.

The first reason is that rapid globalization and environmental changes over the past decade have threatened the sustainability of individuals and societies. Both groups are continuously required to be resilient to overcome the unpredictable global-scale challenges such as complex disasters, economic crises, infectious diseases, terrorism, and social inequality [70].

The second reason is simultaneous adoption of global agreements such as SDGs, PA, and SFDRR in 2015, all of which emphasize *resilience*. The *resilience* perspective has been applied in a broader research field since the mid-2010s, through the strategies of ‘building resilient cities and communities’ on the global agenda related to sustainable development, climate change adaptation, and disaster risk reduction [71,72]. The adoption of *resilience* in national security and local emergency response plans to address unpredictable global threats has sparked growing interest in *resilience thinking* by generating a new *resilience* discourse on government and governance [68]. Consequently, responsibility for emergencies has shifted from the national to the local level in recent years [73,74]. Thus, there is a growing need for *resilience* that overcomes the challenges of complexity, uncertainty, and ambiguity through internal capabilities rather than primitive approaches relying on external interventions.

**Fig. 7:**
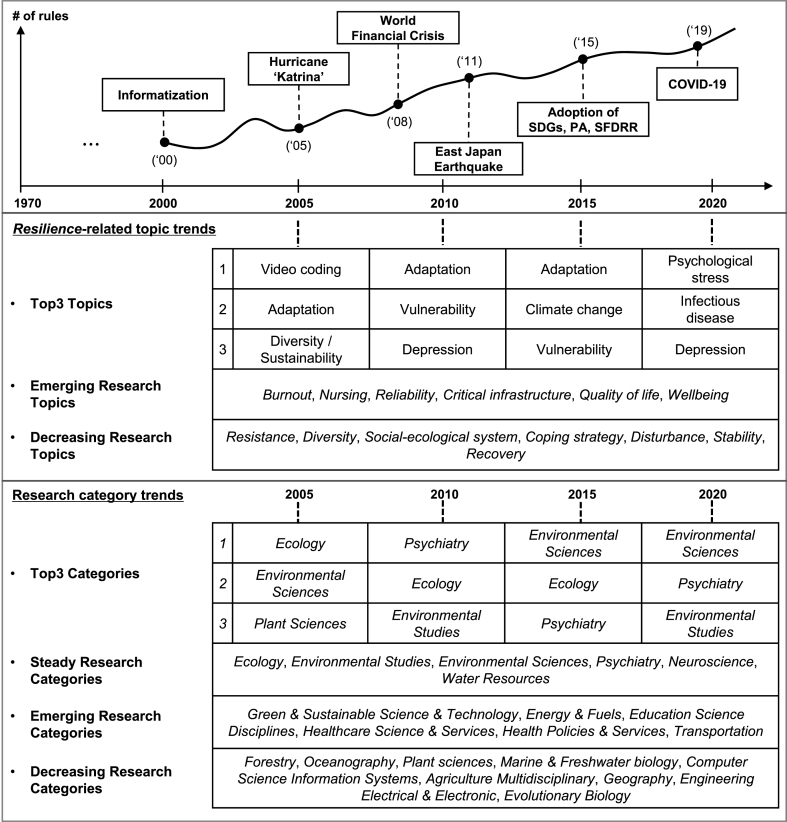
Expansion of *resilience thinking* based on identified research trends.

We illustrate a concept map that describes the expansion of *resilience thinking* along with mega-events and global change (Fig. 7). The *resilience* is better described as a collection of ideas on how to interpret a complex and uncertain world rather than as a well-defined concept [1,53], which is expected to be actively discussed as a new topic in various fields.

## 6 Conclusion

We analyzed the historical progress of *resilience* in terms of co-studied research topics and corresponding research fields using the ARM technique and network analysis. The rule-based approach can provide an in-depth understanding of how the universal term *resilience* has been utilized and interpreted based on a multidisciplinary aspect. The results of this study can have significant implications for scholars worldwide who aim to comprehend *resilience* as a universal norm within their respective research fields. Our study empirically demonstrated the historical progress, diversification and expansion of the concept of *resilience*. While this study is constrained in its ability to predict future prominent topics, its findings can provide valuable insights for researcher seeking to identify *resilience*-related research trend in a world marked by uncertainty and complexity.

The expansion of *resilience thinking* has shown an increasing need for the application of *resilience* as a universal norm or tool for realizing sustainability against uncertain risks and environmental changes. From the perspective of security and disaster management, the dramatic rise in uncertainty and complexity has made the task of anticipating and preventing risks increasingly challenging. In 2010, the United Kingdom (UK)'s National Security Strategy recognized the challenges presented by an “age of uncertainty”. This strategy adopted the concept of *resilience* to decrease country's vulnerability by cultivating *resilience* within the UK. Likewise, the Republic of Korea incorporated the concept of *resilience* in the Fourth Master Plan for National Safety Management (2020–2024) to address inevitability of major disasters, such as the Fukushima nuclear accident in Japan stemming from complex and uncertain risk environments [75].

The global megatrends (i.e., *globalization*) of increasing connectivity and accelerating speed have stimulated the adoption of *resilience* concept in response to the growing complexity and uncertainty. These global changes go beyond risk management and affect overall SES. The unprecedented hyper-connectivity of the SES can accelerate risks that extent beyond seemingly unrelated realms of ecosystems and human life, commonly referred to as *cascading regime* shifts [76]. Advancements in transportation and ICTs have significantly accelerated the speed of communication between diverse individuals and locations, enabling near real-time global connectivity. However, this rapid connectivity and fast speed can create challenges within complex system, as disturbances can propagate uncontrollably, ultimately leading to the collapse of the entire system. The *globalization* has played a significant role in various financial crises and pandemics, including the COVID-19 outbreak. Identifying and responding to issues in such hypercomplex networks, where human society and ecosystems are intricately interconnected and tightly linked, can present significant challenges, thereby increasing risks to everyday life [77,78]. Thus, we find ourselves inhabiting a world characterized by *normal* accidents [78].

The high level of connectivity and speed is not restricted to the environment or engineering systems. In modern society, where everything is connected, people are increasingly exposed to uncertainty. A risk signal can become a global issue with highly connected and globalized media through the social amplification of risk [79]. Uncertainty greatly affects society as a whole and has a profound impact on individual businesses, human relations, health, and psychology. Therefore, *resilience* is a necessary concept in all SES areas. *Resilience* is increasingly expected to be a boundary spanner that promotes communication and collaboration between stakeholders with different interests and scientific knowledge.

## Author contribution statement

Seunghoo Jeong; Cheongil Kim: Conceived and designed the experiments; Analyzed and interpreted the data; Wrote the paper.

Jaesun Yeom: Performed the experiments; Analyzed and interpreted the data; Wrote the paper.

Ji-Bum Chung: Analyzed and interpreted the data; Contributed reagents, materials, analysis tools or data; Wrote the paper.

## Data availability statement

Data included in article/supplementary material/referenced in article.

## Additional information

Supplementary content related to this article has been published online at [URL].

## Declaration of competing interest

The authors declare that they have no known competing financial interests or personal relationships that could have appeared to influence the work reported in this paper.
