# Supplementary material for: Resilience and social change: Findings from research trends using association rule mining
Source: Heliyon. 2023 Jul 27;9(8):e18766. doi: 10.1016/j.heliyon.2023.e18766 (PMC10404750; doi:10.1016/j.heliyon.2023.e18766)
Supplement: Multimedia component 1 [file mmc1.docx]

Appendix A. Yearly rule-based research topics with ARM indices (sorted by support larger than 0.01)

( /: Separated rules)

A.1. 2001~2004 (Sup. = Support, Conf. = Confidence)

| Rank | 2001 | | | | 2002 | | | | 2003 | | | | 2004 | | | |
| --- | --- | --- | --- | --- | --- | --- | --- | --- | --- | --- | --- | --- | --- | --- | --- | --- |
|  | Topics | Sup. | Conf. | Lift | Topics | Sup. | Conf. | Lift | Topics | Sup. | Conf. | Lift | Topics | Sup. | Conf. | Lift |
| 1 | stability | 0.102 | 0.857 | 1.076 | video coding | 0.125 | 0.857 | 1.176 | resistance /  diversity | 0.071 | 1.000 | 1.273 | resistance | 0.087 | 1.000 | 1.195 |
| 2 | resistance | 0.068 | 1.000 | 1.255 |  |  |  |  | psychological stress | 0.057 | 1.000 | 1.273 | stability | 0.076 | 0.875 | 1.045 |
| 3 |  |  |  |  |  |  |  |  |  |  |  |  | vulnerability | 0.065 | 1.000 | 1.195 |
| 4 |  |  |  |  |  |  |  |  |  |  |  |  | disturbance /  diversity | 0.054 | 1.000 | 1.195 |
| 5 |  |  |  |  |  |  |  |  |  |  |  |  | adolescence /  sustainability /  video coding | 0.043 | 1.000 | 1.195 |

A.2. 2005~2008 (Sup. = Support, Conf. = Confidence)

| Rank | 2005 | | | | 2006 | | | | 2007 | | | | 2008 | | | |
| --- | --- | --- | --- | --- | --- | --- | --- | --- | --- | --- | --- | --- | --- | --- | --- | --- |
|  | Topics | Sup. | Conf. | Lift | Topics | Sup. | Conf. | Lift | Topics | Sup. | Conf. | Lift | Topics | Sup. | Conf. | Lift |
| 1 | video coding | 0.071 | 1.000 | 1.217 | adaptation | 0.085 | 0.867 | 1.061 | vulnerability /  video coding | 0.051 | 1.000 | 1.222 | ecosystem | 0.070 | 0.778 | 1.017 |
| 2 | adaptation | 0.063 | 1.000 | 1.217 | social-ecological system /  video coding | 0.065 | 0.909 | 1.113 | sustainability /  adaptation | 0.045 | 1.000 | 1.222 | climate change | 0.060 | 0.857 | 1.120 |
| 3 | diversity /  sustainability | 0.054 | 1.000 | 1.217 | vulnerability | 0.052 | 1.000 | 1.224 | adolescence | 0.040 | 1.000 | 1.222 | vulnerability | 0.045 | 1.000 | 1.307 |
| 4 | disturbance | 0.045 | 1.000 | 1.217 | disturbance | 0.046 | 1.000 | 1.224 | resistance /  psychological stress /  adaptive management | 0.028 | 1.000 | 1.222 | disturbance | 0.040 | 1.000 | 1.307 |
| 5 | stability /  resistance | 0.045 | 0.833 | 1.014 | adaptation, vulnerability | 0.039 | 1.000 | 1.224 | climate change | 0.028 | 0.833 | 1.019 | diversity | 0.040 | 0.889 | 1.162 |
| 6 | social-ecological system /  recovery | 0.036 | 1.000 | 1.217 | trauma /  governance | 0.033 | 1.000 | 1.224 | simulation /  risk /  hysteresis / disturbance /  restoration /  stability | 0.023 | 1.000 | 1.222 | social-ecological system | 0.035 | 1.000 | 1.307 |
| 7 |  |  |  |  | stability /  resistance /  threshold | 0.026 | 1.000 | 1.224 | adaptation, vulnerability /  children /  qualitative research /  mental health /  development /  recovery /  community /  regime shift /  ecosystem management | 0.017 | 1.000 | 1.222 | meteorological disaster | 0.030 | 0.857 | 1.120 |
| 8 |  |  |  |  |  |  |  |  |  |  |  |  | disaster /  recovery /  sustainability /  resistance | 0.025 | 1.000 | 1.307 |
| 9 |  |  |  |  |  |  |  |  |  |  |  |  | depression /  adaptation | 0.025 | 0.833 | 1.089 |
| 10 |  |  |  |  |  |  |  |  |  |  |  |  | ecosystem, climate change /  diversity, climate change | 0.020 | 1.000 | 1.307 |
| 11 |  |  |  |  |  |  |  |  |  |  |  |  | diversity, ecosystem /  video coding | 0.020 | 0.800 | 1.046 |
| 12 |  |  |  |  |  |  |  |  |  |  |  |  | vulnerability, ecosystem /  marginalized people /  recruitment /  ecosystem, disturbance /  climate change, disturbance /  scale /  community /  mental health /  regime shift /  data processing /  forest management | 0.015 | 1.000 | 1.307 |

A.3. 2009~2012 (Sup. = Support, Conf. = Confidence)

| Rank | 2009 | | | | 2010 | | | | 2011 | | | | 2012 | | | |
| --- | --- | --- | --- | --- | --- | --- | --- | --- | --- | --- | --- | --- | --- | --- | --- | --- |
|  | Topics | Sup. | Conf. | Lift | Topics | Sup. | Conf. | Lift | Topics | Sup. | Conf. | Lift | Topics | Sup. | Conf. | Lift |
| 1 | ecosystem | 0.066 | 0.850 | 1.097 | adaptation | 0.056 | 0.857 | 1.137 | vulnerability | 0.075 | 0.914 | 1.156 | adaptation /  climate change | 0.065 | 0.857 | 1.166 |
| 2 | trauma | 0.050 | 1.000 | 1.290 | vulnerability | 0.047 | 1.000 | 1.326 | climate change | 0.073 | 0.816 | 1.032 | vulnerability | 0.059 | 1.000 | 1.360 |
| 3 | adaptation | 0.047 | 0.923 | 1.191 | depression | 0.047 | 0.882 | 1.170 | adaptation | 0.059 | 0.926 | 1.171 | psychological stress | 0.046 | 0.875 | 1.190 |
| 4 | psychological stress | 0.039 | 0.909 | 1.173 | resistance | 0.040 | 1.000 | 1.326 | meteorological disaster | 0.038 | 0.941 | 1.190 | trauma | 0.039 | 1.000 | 1.360 |
| 5 | resistance | 0.035 | 1.000 | 1.290 | psychological stress | 0.040 | 0.929 | 1.232 | trauma /  social-ecological system | 0.038 | 0.889 | 1.124 | resistance | 0.030 | 1.000 | 1.360 |
| 6 | mental health /  regime shift /  stability /  vulnerability | 0.023 | 1.000 | 1.290 | coping strategy | 0.031 | 0.909 | 1.206 | risk | 0.035 | 1.000 | 1.265 | disturbance | 0.024 | 0.917 | 1.247 |
| 7 | meteorological disaster | 0.023 | 0.857 | 1.106 | trauma | 0.031 | 0.769 | 1.020 | psychological stress /  video coding | 0.028 | 1.000 | 1.265 | mental health | 0.024 | 0.846 | 1.151 |
| 8 | adaptation, climate change /  recovery /  anxiety /  social-ecological system /  sustainability /  protected areas | 0.019 | 1.000 | 1.290 | diversity | 0.025 | 0.800 | 1.061 | resistance | 0.028 | 0.923 | 1.168 | depression | 0.024 | 0.786 | 1.068 |
| 9 | governance /  forest | 0.016 | 1.000 | 1.290 | disturbance | 0.022 | 1.000 | 1.326 | mental health /  disturbance /  depression | 0.021 | 0.900 | 1.138 | adaptation, climate change | 0.022 | 0.833 | 1.133 |
| 10 |  |  |  |  | adolescence | 0.022 | 0.875 | 1.161 | governance | 0.021 | 0.818 | 1.035 | coping strategy | 0.020 | 0.900 | 1.224 |
| 11 |  |  |  |  | community /  family / risk /  meteorological disaster | 0.019 | 1.000 | 1.326 | adaptation, climate change | 0.019 | 1.000 | 1.265 | quality of life /  meteorological disaster | 0.017 | 1.000 | 1.360 |
| 12 |  |  |  |  | social-ecological system /  video coding | 0.019 | 0.857 | 1.137 | adolescence | 0.019 | 0.889 | 1.124 | social-ecological system | 0.017 | 0.889 | 1.209 |
| 13 |  |  |  |  | psychological stress, depression /  culture /  management /  stability /  herbivory /  social support | 0.016 | 1.000 | 1.326 | protection | 0.016 | 1.000 | 1.265 | uncertainty | 0.015 | 1.000 | 1.360 |
| 14 |  |  |  |  | psychology /  chronic disease | 0.016 | 0.833 | 1.105 | adaptation, vulnerability | 0.016 | 0.875 | 1.107 | chronic disease | 0.015 | 0.778 | 1.058 |
| 15 |  |  |  |  | adaptation, climate change /  threshold /  diversity, ecosystem /  hazard | 0.012 | 1.000 | 1.326 | risk, vulnerability /  disaster /  uncertainty /  vulnerability, meteorological disaster /  quality of life /  coping strategy / water management | 0.014 | 1.000 | 1.265 | qualitative research | 0.013 | 1.000 | 1.360 |
| 16 |  |  |  |  | depression, trauma /  restoration | 0.012 | 0.800 | 1.061 | scale /  complexity theory / stability | 0.012 | 1.000 | 1.265 | risk management | 0.013 | 0.857 | 1.166 |
| 17 |  |  |  |  |  |  |  |  | ecosystem, disturbance /  anxiety | 0.012 | 0.833 | 1.054 | governance /  sustainability | 0.013 | 0.750 | 1.020 |
| 18 |  |  |  |  |  |  |  |  |  |  |  |  | adaptation, vulnerability /  personality | 0.011 | 1.000 | 1.360 |
| 19 |  |  |  |  |  |  |  |  |  |  |  |  | critical infrastructure /  robustness /  nursing / risk /  social capital | 0.011 | 0.833 | 1.133 |

A.4. 2013~2016 (Sup. = Support, Conf. = Confidence)

| Rank | 2013 | | | | 2014 | | | | 2015 | | | | 2016 | | | |
| --- | --- | --- | --- | --- | --- | --- | --- | --- | --- | --- | --- | --- | --- | --- | --- | --- |
|  | Topics | Sup. | Conf. | Lift | Topics | Sup. | Conf. | Lift | Topics | Sup. | Conf. | Lift | Topics | Sup. | Conf. | Lift |
| 1 | climate change | 0.043 | 0.727 | 1.015 | climate change | 0.054 | 0.769 | 1.061 | adaptation | 0.066 | 0.906 | 1.253 | adaptation | 0.049 | 0.731 | 1.048 |
| 2 | psychological stress | 0.041 | 0.958 | 1.337 | trauma | 0.038 | 0.903 | 1.246 | climate change | 0.062 | 0.740 | 1.023 | climate change | 0.044 | 0.743 | 1.066 |
| 3 | depression | 0.037 | 0.955 | 1.332 | vulnerability | 0.038 | 0.824 | 1.136 | vulnerability | 0.041 | 0.900 | 1.244 | vulnerability | 0.039 | 0.902 | 1.294 |
| 4 | trauma | 0.032 | 0.900 | 1.256 | psychological stress | 0.037 | 0.931 | 1.285 | sustainability | 0.039 | 0.810 | 1.119 | sustainability | 0.035 | 0.820 | 1.176 |
| 5 | sustainability | 0.032 | 0.750 | 1.047 | depression | 0.035 | 0.813 | 1.121 | psychological stress | 0.036 | 0.970 | 1.341 | depression | 0.027 | 0.889 | 1.275 |
| 6 | diversity | 0.030 | 0.850 | 1.186 | sustainability | 0.029 | 0.778 | 1.073 | depression | 0.027 | 0.960 | 1.327 | trauma | 0.027 | 0.886 | 1.270 |
| 7 | resistance | 0.029 | 1.000 | 1.396 | resistance | 0.026 | 1.000 | 1.380 | resistance | 0.026 | 1.000 | 1.383 | psychological stress | 0.026 | 0.811 | 1.163 |
| 8 | social-ecological system | 0.027 | 0.938 | 1.308 | social-ecological system | 0.026 | 0.826 | 1.140 | trauma | 0.026 | 0.885 | 1.223 | meteorological disaster | 0.023 | 0.711 | 1.019 |
| 9 | mental health | 0.023 | 0.867 | 1.209 | Risk | 0.019 | 0.933 | 1.288 | risk management | 0.026 | 0.793 | 1.097 | resistance | 0.021 | 0.926 | 1.328 |
| 10 | governance | 0.021 | 1.000 | 1.396 | disaster | 0.019 | 0.875 | 1.207 | adaptation, climate change | 0.025 | 1.000 | 1.383 | recovery | 0.021 | 0.893 | 1.281 |
| 11 | risk /  anxiety | 0.020 | 0.917 | 1.279 | diversity | 0.018 | 0.867 | 1.196 | adolescence | 0.022 | 1.000 | 1.383 | diversity | 0.020 | 0.885 | 1.269 |
| 12 | recovery /  disturbance | 0.020 | 0.846 | 1.181 | climate change, adaptation | 0.018 | 0.765 | 1.055 | coping strategy | 0.022 | 0.950 | 1.314 | mental health | 0.019 | 0.880 | 1.262 |
| 13 | meteorological disaster | 0.020 | 0.733 | 1.023 | recovery /  adolescence | 0.016 | 0.923 | 1.274 | disturbance | 0.019 | 1.000 | 1.383 | risk | 0.018 | 0.875 | 1.255 |
| 14 | regime shift /  adolescence /  psychological stress, trauma | 0.016 | 1.000 | 1.396 | mental health | 0.016 | 0.857 | 1.183 | mental health | 0.019 | 0.850 | 1.175 | social-ecological system | 0.018 | 0.778 | 1.116 |
| 15 | adaptive management /  coping strategy /  stability | 0.012 | 0.875 | 1.221 | anxiety | 0.015 | 0.917 | 1.265 | anxiety /  recovery | 0.017 | 0.938 | 1.296 | risk management | 0.016 | 0.760 | 1.090 |
| 16 | education /  psychological stress, depression | 0.011 | 1.000 | 1.396 | built environment | 0.014 | 1.000 | 1.380 | stability | 0.017 | 0.882 | 1.220 | nursing | 0.015 | 0.857 | 1.229 |
| 17 | australia /  risk management | 0.011 | 0.750 | 1.047 | governance | 0.014 | 0.909 | 1.254 | disaster | 0.016 | 0.933 | 1.290 | critical infrastructure | 0.015 | 0.750 | 1.076 |
| 18 |  |  |  |  | restoration | 0.012 | 0.900 | 1.242 | risk | 0.015 | 0.867 | 1.198 | disturbance | 0.015 | 0.944 | 1.355 |
| 19 |  |  |  |  | stability /  reliability | 0.012 | 0.818 | 1.129 | critical infrastructure | 0.014 | 0.923 | 1.276 | quality of life | 0.014 | 1.000 | 1.434 |
| 20 |  |  |  |  | cancer /  critical infrastructure | 0.011 | 1.000 | 1.380 | marginalized people | 0.014 | 0.800 | 1.106 | coping strategy | 0.013 | 0.882 | 1.266 |
| 21 |  |  |  |  | coping strategy / robustness /  complex system | 0.011 | 0.889 | 1.226 | redundancy | 0.013 | 0.786 | 1.086 | governance | 0.013 | 0.714 | 1.025 |
| 22 |  |  |  |  | conservation / disturbance | 0.011 | 0.800 | 1.104 | uncertainty /  depression, anxiety | 0.010 | 0.900 | 1.244 | adolescence | 0.012 | 0.875 | 1.255 |
| 23 |  |  |  |  |  |  |  |  |  |  |  |  | adaptation, climate change | 0.012 | 0.778 | 1.116 |
| 24 |  |  |  |  |  |  |  |  |  |  |  |  | wellbeing | 0.011 | 1.000 | 1.434 |
| 25 |  |  |  |  |  |  |  |  |  |  |  |  | community | 0.011 | 0.929 | 1.332 |
| 26 |  |  |  |  |  |  |  |  |  |  |  |  | social support | 0.011 | 0.867 | 1.243 |
| 27 |  |  |  |  |  |  |  |  |  |  |  |  | stability | 0.010 | 0.857 | 1.229 |

A.5. 2017~2020 (Sup. = Support, Conf. = Confidence)

| Rank | 2017 | | | | 2018 | | | | 2019 | | | | 2020 | | | |
| --- | --- | --- | --- | --- | --- | --- | --- | --- | --- | --- | --- | --- | --- | --- | --- | --- |
|  | Topics | Sup. | Conf. | Lift | Topics | Sup. | Conf. | Lift | Topics | Sup. | Conf. | Lift | Topics | Sup. | Conf. | Lift |
| 1 | climate change | 0.057 | 0.737 | 1.072 | climate change | 0.048 | 0.681 | 1.018 | adaptation | 0.028 | 0.697 | 1.130 | psychological stress | 0.035 | 0.874 | 1.345 |
| 2 | adaptation | 0.040 | 0.831 | 1.208 | adaptation | 0.042 | 0.791 | 1.181 | mental health | 0.027 | 0.836 | 1.355 | infectious disease | 0.033 | 0.810 | 1.246 |
| 3 | depression | 0.035 | 0.896 | 1.304 | vulnerability | 0.039 | 0.925 | 1.382 | meteorological disaster | 0.026 | 0.681 | 1.103 | depression | 0.032 | 0.882 | 1.357 |
| 4 | sustainability | 0.031 | 0.809 | 1.176 | psychological stress | 0.033 | 0.869 | 1.298 | vulnerability | 0.024 | 0.865 | 1.402 | vulnerability | 0.031 | 0.899 | 1.384 |
| 5 | vulnerability | 0.030 | 0.974 | 1.417 | trauma | 0.028 | 0.957 | 1.430 | depression | 0.023 | 0.878 | 1.422 | sustainability | 0.028 | 0.857 | 1.319 |
| 6 | psychological stress | 0.028 | 0.872 | 1.269 | depression | 0.027 | 0.843 | 1.259 | psychological stress | 0.021 | 0.830 | 1.345 | adaptation | 0.028 | 0.706 | 1.087 |
| 7 | mental health | 0.027 | 0.971 | 1.412 | mental health | 0.026 | 0.854 | 1.276 | resistance | 0.020 | 0.974 | 1.578 | mental health | 0.024 | 0.763 | 1.174 |
| 8 | trauma | 0.027 | 0.943 | 1.372 | sustainability | 0.021 | 0.733 | 1.095 | sustainability | 0.019 | 0.632 | 1.023 | trauma | 0.022 | 0.933 | 1.437 |
| 9 | resistance | 0.019 | 0.920 | 1.339 | recovery | 0.017 | 0.875 | 1.307 | trauma | 0.018 | 0.919 | 1.489 | burnout | 0.019 | 0.980 | 1.508 |
| 10 | recovery | 0.019 | 0.852 | 1.240 | resistance | 0.016 | 0.963 | 1.438 | recovery | 0.017 | 0.861 | 1.395 | adolescence | 0.015 | 0.951 | 1.464 |
| 11 | coping strategy | 0.018 | 0.917 | 1.334 | social support | 0.016 | 0.929 | 1.387 | burnout | 0.016 | 0.967 | 1.567 | anxiety | 0.015 | 0.864 | 1.329 |
| 12 | critical infrastructure | 0.018 | 0.759 | 1.104 | diversity | 0.016 | 0.839 | 1.253 | adolescence | 0.016 | 0.906 | 1.469 | risk | 0.015 | 0.841 | 1.294 |
| 13 | anxiety | 0.015 | 0.947 | 1.378 | disturbance | 0.016 | 0.862 | 1.287 | quality of life | 0.016 | 0.853 | 1.382 | resistance | 0.013 | 0.944 | 1.454 |
| 14 | adolescence | 0.014 | 0.850 | 1.237 | adaptation, climate change | 0.014 | 0.846 | 1.264 | diversity | 0.014 | 0.765 | 1.239 | critical infrastructure | 0.013 | 0.708 | 1.090 |
| 15 | diversity | 0.014 | 0.708 | 1.031 | disaster | 0.014 | 0.710 | 1.060 | risk | 0.013 | 0.889 | 1.440 | nursing | 0.013 | 0.825 | 1.270 |
| 16 | risk /  reliability | 0.013 | 0.941 | 1.369 | reliability | 0.012 | 0.870 | 1.299 | wellbeing | 0.012 | 0.885 | 1.434 | coping strategy | 0.013 | 0.889 | 1.368 |
| 17 | quality of life | 0.013 | 0.800 | 1.164 | social-ecological system | 0.012 | 0.741 | 1.106 | disturbance | 0.012 | 0.852 | 1.380 | optimization | 0.013 | 0.865 | 1.331 |
| 18 | wellbeing | 0.011 | 0.933 | 1.358 | burnout /  nursing | 0.012 | 0.864 | 1.290 | nursing | 0.011 | 0.955 | 1.547 | reliability | 0.012 | 0.816 | 1.256 |
| 19 | nursing /  disaster | 0.011 | 0.875 | 1.273 | coping strategy | 0.011 | 0.850 | 1.269 |  |  |  |  | quality of life | 0.012 | 0.857 | 1.319 |
| 20 | disturbance | 0.011 | 0.778 | 1.132 | wellbeing | 0.011 | 0.810 | 1.209 |  |  |  |  | diversity | 0.011 | 0.853 | 1.313 |
| 21 | robustness | 0.011 | 0.929 | 1.351 |  |  |  |  |  |  |  |  | disaster | 0.011 | 0.744 | 1.145 |
| 22 | social-ecological system | 0.011 | 0.765 | 1.113 |  |  |  |  |  |  |  |  | microgrid | 0.011 | 0.737 | 1.134 |
| 23 |  |  |  |  |  |  |  |  |  |  |  |  | recovery | 0.011 | 0.794 | 1.222 |
| 24 |  |  |  |  |  |  |  |  |  |  |  |  | adaptation, climate change | 0.010 | 0.703 | 1.082 |

Appendix B. Yearly ranking of rule-based research topics (sorted by alphabetical order)

| **Research Topics** | **‘01** | **‘02** | **‘03** | **‘04** | **‘05** | **‘06** | **‘07** | **‘08** | **‘09** | **‘10** | **‘11** | **‘12** | **‘13** | **‘14** | **‘15** | **‘16** | **‘17** | **‘18** | **‘19** | **‘20** |
| --- | --- | --- | --- | --- | --- | --- | --- | --- | --- | --- | --- | --- | --- | --- | --- | --- | --- | --- | --- | --- |
| adaptation | - | - | - | - | 2 | 1 | 2 | 9 | 3 | 1 | 3 | 1 | - | - | 1 | 1 | 2 | 2 | 1 | 6 |
| adaptation, climate change | - | - | - | - | - | - | - | - | 8 | 15 | 11 | 9 | - | 12 | 10 | 23 | - | 14 | - | 24 |
| adaptation, vulnerability | - | - | - | - | - | 5 | 7 | - | - | - | 14 | 18 | - | - | - | - | - | - | - | - |
| adaptive management | - | - | - | - | - | - | 4 | - | - | - | - | - | 15 | - | - | - | - | - | - | - |
| adolescence | - | - | - | 5 | - | - | 3 | - | - | 10 | 12 | - | 14 | 13 | 11 | 22 | 14 | - | 12 | 10 |
| anxiety | - | - | - | - | - | - | - | - | 8 | - | 17 | - | 11 | 15 | 15 | - | 13 | - | - | 11 |
| australia | - | - | - | - | - | - | - | - | - | - | - | - | 17 | - | - | - | - | - | - | - |
| built environment | - | - | - | - | - | - | - | - | - | - | - | - | - | 16 | - | - | - | - | - | - |
| burnout | - | - | - | - | - | - | - | - | - | - | - | - | - | - | - | - | - | 18 | 11 | 9 |
| cancer | - | - | - | - | - | - | - | - | - | - | - | - | - | 20 | - | - | - | - | - | - |
| children | - | - | - | - | - | - | 7 | - | - | - | - | - | - | - | - | - | - | - | - | - |
| chronic disease | - | - | - | - | - | - | - | - | - | 14 | - | 14 | - | - | - | - | - | - | - | - |
| climate change | - | - | - | - | - | - | 5 | 2 | - | - | 2 | 1 | 1 | 1 | 2 | 2 | 1 | 1 | - | - |
| climate change, disturbance | - | - | - | - | - | - | - | 12 | - | - | - | - | - | - | - | - | - | - | - | - |
| community | - | - | - | - | - | - | 7 | 12 | - | 11 | - | - | - | - | - | 25 | - | - | - | - |
| complex system | - | - | - | - | - | - | - | - | - | - | - | - | - | 21 | - | - | - | - | - | - |
| complexity theory | - | - | - | - | - | - | - | - | - | - | 16 | - | - | - | - | - | - | - | - | - |
| conservation | - | - | - | - | - | - | - | - | - | - | - | - | - | 22 | - | - | - | - | - | - |
| coping strategy | - | - | - | - | - | - | - | - | - | 6 | 15 | 10 | 15 | 21 | 12 | 20 | 11 | 19 | - | 16 |
| critical infrastructure | - | - | - | - | - | - | - | - | - | - | - | 19 | - | 20 | 19 | 17 | 12 | - | - | 14 |
| culture | - | - | - | - | - | - | - | - | - | 13 | - | - | - | - | - | - | - | - | - | - |
| data processing | - | - | - | - | - | - | - | 12 | - | - | - | - | - | - | - | - | - | - | - | - |
| depression | - | - | - | - | - | - | - | 9 | - | 3 | 9 | 8 | 3 | 5 | 6 | 5 | 3 | 6 | 5 | 3 |
| depression, anxiety | - | - | - | - | - | - | - | - | - | - | - | - | - | - | 22 | - | - | - | - | - |
| depression, psychological stress | - | - | - | - | - | - | - | - | - | 13 | - | - | - | - | - | - | - | - | - | - |
| depression, trauma | - | - | - | - | - | - | - | - | - | 16 | - | - | - | - | - | - | - | - | - | - |
| development | - | - | - | - | - | - | 7 | - | - | - | - | - | - | - | - | - | - | - | - | - |
| disaster | - | - | - | - | - | - | - | 8 | - | - | 15 | - | - | 10 | 17 | - | 19 | 15 | - | 21 |
| disturbance | - | - | - | 4 | 4 | 4 | 6 | 4 | - | 9 | 9 | 6 | 12 | 22 | 13 | 18 | 20 | 13 | 17 | - |
| diversity | - | - | 1 | 4 | 3 | - | - | 5 | - | 8 | - | - | 6 | 11 | - | 11 | 15 | 12 | 14 | 20 |
| diversity, climate change | - | - | - | - | - | - | - | 10 | - | - | - | - | - | - | - | - | - | - | - | - |
| diversity, ecosystem | - | - | - | - | - | - | - | 11 | - | 15 | - | - | - | - | - | - | - | - | - | - |
| ecosystem | - | - | - | - | - | - | - | 1 | 1 | - | - | - | - | - | - | - | - | - | - | - |
| ecosystem management | - | - | - | - | - | - | 7 | - | - | - | - | - | - | - | - | - | - | - | - | - |
| ecosystem, climate change | - | - | - | - | - | - | - | 10 | - | - | - | - | - | - | - | - | - | - | - | - |
| ecosystem, disturbance | - | - | - | - | - | - | - | 12 | - | - | 17 | - | - | - | - | - | - | - | - | - |
| education | - | - | - | - | - | - | - | - | - | - | - | - | 16 | - | - | - | - | - | - | - |
| family | - | - | - | - | - | - | - | - | - | 11 | - | - | - | - | - | - | - | - | - | - |
| forest | - | - | - | - | - | - | - | - | 9 | - | - | - | - | - | - | - | - | - | - | - |
| forest management | - | - | - | - | - | - | - | 12 | - | - | - | - | - | - | - | - | - | - | - | - |
| governance | - | - | - | - | - | 6 | - | - | 9 | - | 10 | 17 | 10 | 17 | - | 21 | - | - | - | - |
| hazard | - | - | - | - | - | - | - | - | - | 15 | - | - | - | - | - | - | - | - | - | - |
| herbivory | - | - | - | - | - | - | - | - | - | 13 | - | - | - | - | - | - | - | - | - | - |
| hysteresis | - | - | - | - | - | - | 6 | - | - | - | - | - | - | - | - | - | - | - | - | - |
| infectious disease | - | - | - | - | - | - | - | - | - | - | - | - | - | - | - | - | - | - | - | 2 |
| management | - | - | - | - | - | - | - | - | - | 13 | - | - | - | - | - | - | - | - | - | - |
| marginalized people | - | - | - | - | - | - | - | 12 | - | - | - | - | - | - | 20 | - | - | - | - | - |
| mental health | - | - | - | - | - | - | 7 | 12 | 6 | - | 9 | 7 | 9 | 14 | 14 | 12 | 7 | 7 | 2 | 7 |
| meteorological disaster | - | - | - | - | - | - | - | 7 | 7 | 11 | 4 | 11 | 13 | - | - | 8 | - | - | 3 | - |
| microgrid | - | - | - | - | - | - | - | - | - | - | - | - | - | - | - | - | - | - | - | 22 |
| nursing | - | - | - | - | - | - | - | - | - | - | - | 19 | - | - | - | 16 | 19 | 18 | 18 | 15 |
| optimization | - | - | - | - | - | - | - | - | - | - | - | - | - | - | - | - | - | - | - | 17 |
| personality | - | - | - | - | - | - | - | - | - | - | - | 18 | - | - | - | - | - | - | - | - |
| protected areas | - | - | - | - | - | - | - | - | 8 | - | - | - | - | - | - | - | - | - | - | - |
| protection | - | - | - | - | - | - | - | - | - | - | 13 | - | - | - | - | - | - | - | - | - |
| psychological stress | - | - | 2 | - | - | - | 4 | - | 4 | 5 | 7 | 3 | 2 | 4 | 5 | 7 | 6 | 4 | 6 | 1 |
| psychological stress, depression | - | - | - | - | - | - | - | - | - | - | - | - | 16 | - | - | - | - | - | - | - |
| psychological stress, trauma | - | - | - | - | - | - | - | - | - | - | - | - | 14 | - | - | - | - | - | - | - |
| psychology | - | - | - | - | - | - | - | - | - | 14 | - | - | - | - | - | - | - | - | - | - |
| qualitative research | - | - | - | - | - | - | 7 | - | - | - | - | 15 | - | - | - | - | - | - | - | - |
| quality of life | - | - | - | - | - | - | - | - | - | - | 15 | 11 | - | - | - | 19 | 17 | - | 13 | 19 |
| recovery | - | - | - | - | 6 | - | 7 | 8 | 8 | - | - | - | 12 | 13 | 15 | 10 | 10 | 9 | 10 | 23 |
| recruitment | - | - | - | - | - | - | - | 12 | - | - | - | - | - | - | - | - | - | - | - | - |
| redundancy | - | - | - | - | - | - | - | - | - | - | - | - | - | - | 21 | - | - | - | - | - |
| regime shift | - | - | - | - | - | - | 7 | 12 | 6 | - | - | - | 14 | - | - | - | - | - | - | - |
| reliability | - | - | - | - | - | - | - | - | - | - | - | - | - | 19 | - | - | 16 | 16 | - | 18 |
| resistance | 2 | - | 1 | 1 | 5 | 7 | 4 | 8 | 5 | 4 | 8 | 5 | 7 | 7 | 7 | 9 | 9 | 10 | 7 | 13 |
| restoration | - | - | - | - | - | - | 6 | - | - | 16 | - | - | - | 18 | - | - | - | - | - | - |
| risk | - | - | - | - | - | - | 6 | - | - | 11 | 6 | 19 | 11 | 9 | 18 | 13 | 16 | - | 15 | 12 |
| risk management | - | - | - | - | - | - | - | - | - | - | - | 16 | 17 | - | 9 | 15 | - | - | - | - |
| risk, vulnerability | - | - | - | - | - | - | - | - | - | - | 15 | - | - | - | - | - | - | - | - | - |
| robustness | - | - | - | - | - | - | - | - | - | - | - | 19 | - | 21 | - | - | 21 | - | - | - |
| scale | - | - | - | - | - | - | - | 12 | - | - | 16 | - | - | - | - | - | - | - | - | - |
| simulation | - | - | - | - | - | - | 6 | - | - | - | - | - | - | - | - | - | - | - | - | - |
| social capital | - | - | - | - | - | - | - | - | - | - | - | 19 | - | - | - | - | - | - | - | - |
| social support | - | - | - | - | - | - | - | - | - | 13 | - | - | - | - | - | 26 | - | 11 | - | - |
| social-ecological system | - | - | - | - | 6 | 2 | - | 6 | 8 | 12 | 5 | 12 | 8 | 8 | - | 14 | 22 | 17 | - | - |
| stability | 1 | - | - | 2 | 5 | 7 | 6 | - | 6 | 13 | 16 | - | 15 | 19 | 16 | 27 | - | - | - | - |
| sustainability | - | - | - | 5 | 3 | - | 2 | 8 | 8 | - | - | 17 | 5 | 6 | 4 | 4 | 4 | 8 | 8 | 5 |
| threshold | - | - | - | - | - | 7 | - | - | - | 15 | - | - | - | - | - | - | - | - | - | - |
| trauma | - | - | - | - | - | 6 | - | - | 2 | 7 | 5 | 4 | 4 | 2 | 8 | 6 | 8 | 5 | 9 | 8 |
| uncertainty | - | - | - | - | - | - | - | - | - | - | 15 | 13 | - | - | 22 | - | - | - | - | - |
| video coding | - | 1 | - | 5 | 1 | 2 | 1 | 11 | - | 12 | 7 | - | - | - | - | - | - | - | - | - |
| vulnerability | - | - | - | 3 | - | 3 | 1 | 3 | 6 | 2 | 1 | 2 | - | 3 | 3 | 3 | 5 | 3 | 4 | 4 |
| vulnerability, ecosystem | - | - | - | - | - | - | - | 12 | - | - | - | - | - | - | - | - | - | - | - | - |
| vulnerability, meteorological disaster | - | - | - | - | - | - | - | - | - | - | 15 | - | - | - | - | - | - | - | - | - |
| water management | - | - | - | - | - | - | - | - | - | - | 15 | - | - | - | - | - | - | - | - | - |
| wellbeing | - | - | - | - | - | - | - | - | - | - | - | - | - | - | - | 24 | 18 | 20 | 16 | - |

Appendix C. Yearly ranking of research categories (sorted by alphabetical order)

| **Research Categories** | **‘01** | **‘02** | **‘03** | **‘04** | **‘05** | **‘06** | **‘07** | **‘08** | **‘09** | **‘10** | **‘11** | **‘12** | **‘13** | **‘14** | **‘15** | **‘16** | **‘17** | **‘18** | **‘19** | **‘20** |
| --- | --- | --- | --- | --- | --- | --- | --- | --- | --- | --- | --- | --- | --- | --- | --- | --- | --- | --- | --- | --- |
| Agricultural Economics & Policy | - | - | - | - | - | - | - | - | - | - | 21 | - | - | 25 | 29 | - | - | - | - | 39 |
| Agricultural Engineering | - | - | - | - | - | - | - | - | - | 17 | - | 20 | - | - | - | - | - | 26 | - | - |
| Agriculture Dairy & Animal Science | - | - | - | - | 7 | 9 | - | - | 12 | 18 | - | 20 | 21 | 25 | - | 29 | 27 | 26 | - | 35 |
| Agriculture Multidisciplinary | - | - | - | 5 | 6 | 9 | 12 | - | 12 | 17 | - | 14 | 15 | 23 | 15 | 29 | 24 | 20 | 26 | 36 |
| Agronomy | - | - | - | 5 | 5 | - | - | 13 | - | 17 | - | - | - | - | 22 | 23 | 30 | 23 | 23 | 32 |
| Allergy | - | - | - | - | - | - | 13 | - | - | - | - | - | - | - | - | - | - | - | 29 | - |
| Anesthesiology | - | - | - | - | - | - | - | - | - | 18 | - | - | - | - | 29 | - | - | 25 | 27 | 38 |
| Anthropology | - | - | - | - | - | - | - | - | - | - | - | - | - | 25 | 28 | 29 | - | - | - | 37 |
| Archaeology | - | - | - | - | - | - | - | - | - | - | - | - | - | 25 | - | - | - | - | - | 40 |
| Architecture | - | - | - | - | - | - | - | - | - | - | - | - | - | - | - | - | - | 26 | - | - |
| Audiology & Speech-Language Pathology | - | - | - | - | - | - | - | - | - | - | - | - | - | - | 29 | - | - | - | - | - |
| Automation & Control Systems | - | - | - | - | - | - | - | - | - | - | - | - | - | - | 29 | 30 | 31 | 26 | - | 36 |
| Behavioral Sciences | - | - | - | 5 | - | - | - | - | 10 | 15 | 12 | 15 | 13 | 15 | 9 | 19 | 11 | 16 | 24 | 23 |
| Biochemical Research Methods | - | - | - | - | - | - | - | - | - | - | - | - | - | - | - | 29 | - | - | - | - |
| Biochemistry & Molecular Biology | - | - | - | - | - | 9 | 13 | - | 12 | - | 22 | - | 17 | 23 | 29 | 29 | 31 | - | 29 | 34 |
| Biodiversity Conservation | - | - | - | 5 | 6 | 9 | 13 | 8 | 6 | 16 | 13 | 13 | 14 | 8 | 11 | 10 | 17 | 18 | 16 | 17 |
| Biology | 3 | - | - | - | - | - | 13 | 8 | 10 | - | 17 | 19 | 19 | 21 | 20 | 22 | 29 | 22 | 23 | 36 |
| Biophysics | - | - | - | - | - | - | - | - | - | - | - | - | - | - | - | - | - | - | - | 38 |
| Biotechnology & Applied Microbiology | - | - | - | - | - | - | - | - | - | - | 21 | 20 | 21 | 25 | - | - | - | 23 | 28 | 39 |
| Business | - | - | - | - | - | - | - | - | - | - | - | - | - | - | - | - | - | - | 29 | 39 |
| Business Finance | - | - | - | - | - | - | - | - | - | - | - | - | - | - | - | 30 | - | - | - | - |
| Cardiac & Cardiovascular Systems | - | - | - | - | - | - | - | - | 12 | - | - | 20 | - | 24 | - | 27 | 27 | 24 | 27 | 38 |
| Cell Biology | - | - | - | - | - | - | - | - | - | - | - | - | - | - | - | - | 31 | - | - | 39 |
| Chemistry Applied | - | - | - | - | - | - | - | - | - | - | - | 19 | 21 | - | 28 | - | - | - | - | 40 |
| Chemistry Multidisciplinary | - | - | - | - | - | - | - | - | - | - | - | - | - | - | - | 29 | - | - | 28 | 39 |
| Chemistry Physical | - | - | - | - | - | - | - | - | - | - | - | - | - | - | - | - | - | - | 29 | - |
| Clinical Neurology | - | - | - | - | - | 8 | - | 11 | 10 | 7 | 15 | 9 | 7 | 15 | 15 | 15 | 12 | 9 | 10 | 11 |
| Computer Science Artificial Intelligence | - | - | - | - | 7 | - | 12 | - | - | 18 | 22 | 20 | - | - | - | 30 | - | - | - | - |
| Computer Science Cybernetics | - | - | - | - | - | - | - | - | - | - | - | - | - | - | 29 | - | - | - | - | - |
| Computer Science Hardware & Architecture | - | 2 | - | - | - | 9 | - | 13 | - | 18 | 21 | - | - | - | - | - | 29 | 26 | - | 37 |
| Computer Science Information Systems | - | 2 | - | 5 | 5 | 7 | 11 | 10 | - | 18 | 16 | 20 | - | 24 | 28 | 30 | 29 | 22 | 28 | 19 |
| Computer Science Interdisciplinary Applications | - | - | - | - | - | - | 9 | - | - | - | 22 | 20 | 19 | 25 | 27 | - | 28 | - | 24 | 35 |
| Computer Science Software Engineering | - | - | - | 5 | 6 | 8 | 12 | 9 | - | - | 21 | - | - | - | - | - | 31 | 26 | - | 37 |
| Computer Science Theory & Methods | - | - | - | - | 7 | - | - | - | - | - | 21 | - | - | - | - | 30 | 31 | - | - | 39 |
| Construction & Building Technology | - | - | - | - | - | - | - | - | - | - | - | 10 | - | 9 | 16 | 9 | 23 | 20 | 22 | 30 |
| Criminology & Penology | - | - | - | - | - | - | - | - | - | - | - | - | - | - | - | - | - | - | 29 | - |
| Critical Care Medicine | - | - | - | - | - | - | - | - | - | - | - | 19 | 19 | 25 | 29 | 28 | 27 | 26 | 23 | 33 |
| Dentistry Oral Surgery & Medicine | - | - | - | 5 | - | - | - | - | 12 | 18 | - | - | - | - | - | 30 | - | - | 28 | 39 |
| Dermatology | - | - | - | - | - | - | - | - | - | - | - | - | 21 | - | - | 29 | 30 | 26 | 29 | - |
| Developmental Biology | - | - | - | - | - | - | - | - | 12 | 16 | - | - | - | - | - | - | - | 25 | - | 40 |
| Ecology | 1 | - | 1 | 1 | 1 | 2 | 1 | 1 | 1 | 2 | 1 | 3 | 1 | 1 | 2 | 1 | 3 | 3 | 3 | 10 |
| Economics | - | - | - | - | 7 | - | 10 | - | 12 | 18 | 21 | - | 19 | 20 | 26 | 27 | 28 | 22 | 28 | 33 |
| Education & Educational Research | - | - | - | - | - | - | - | - | - | - | - | - | 21 | - | 29 | 30 | - | 24 | 27 | - |
| Education Scientific Disciplines | - | - | - | - | - | - | - | - | - | - | - | 20 | 21 | - | 28 | 26 | 23 | 17 | 23 | 29 |
| Electrochemistry | - | - | - | - | - | - | - | - | - | - | - | - | - | - | - | - | - | - | 29 | - |
| Emergency Medicine | - | - | - | - | - | - | - | - | - | - | - | 19 | 21 | 23 | 28 | 29 | - | 24 | 27 | 36 |
| Endocrinology & Metabolism | - | - | - | - | - | - | 13 | - | - | 14 | 22 | 16 | 14 | 18 | 29 | 29 | 26 | 23 | 20 | 37 |
| Energy & Fuels | - | - | - | - | - | - | - | - | - | 16 | - | 20 | - | 24 | 26 | 19 | 23 | 22 | 20 | 19 |
| Engineering Chemical | - | - | - | - | - | - | - | - | - | - | 22 | - | - | - | - | 28 | 31 | 26 | 28 | 35 |
| Engineering Civil | - | - | - | - | - | 9 | - | - | 12 | 12 | 20 | 13 | 20 | 10 | 8 | 12 | 5 | 17 | 20 | 14 |
| Engineering Electrical & Electronic | - | 1 | - | 3 | 5 | 5 | 8 | 11 | - | 13 | 15 | 20 | 20 | - | 28 | 29 | 23 | 21 | 25 | 8 |
| Engineering Environmental | - | - | - | - | - | - | 9 | - | 12 | 18 | 20 | 13 | 16 | 19 | 21 | 28 | 16 | 18 | 16 | 25 |
| Engineering Geological | - | - | - | - | - | - | - | - | - | - | 20 | - | 21 | 22 | 29 | 27 | 29 | - | - | 40 |
| Engineering Industrial | - | - | - | - | - | - | - | - | - | 18 | 17 | 19 | 20 | 20 | 25 | 21 | 25 | 18 | 17 | 22 |
| Engineering Manufacturing | - | - | - | - | - | - | - | - | - | - | 19 | 20 | - | 24 | - | 29 | 30 | 25 | 28 | 40 |
| Engineering Marine | - | - | - | - | - | - | - | - | - | - | - | - | - | - | - | - | 31 | 24 | 29 | - |
| Engineering Mechanical | - | - | - | - | - | - | - | - | - | - | - | 20 | - | - | - | 28 | 28 | - | 27 | 35 |
| Engineering Multidisciplinary | - | - | - | - | - | - | - | - | - | - | - | 18 | - | 24 | - | 29 | - | 23 | 28 | 35 |
| Engineering Ocean | - | - | - | - | - | - | - | - | - | - | - | - | - | 25 | 29 | 30 | 31 | 24 | 29 | - |
| Entomology | - | - | - | - | - | - | - | - | - | - | - | - | - | - | - | 29 | - | - | 28 | 40 |
| Environmental Sciences | - | - | 2 | 2 | 2 | 3 | 2 | 6 | 2 | 4 | 2 | 1 | 3 | 2 | 1 | 2 | 1 | 1 | 1 | 1 |
| Environmental Studies | - | - | - | 5 | 4 | 1 | 3 | 4 | 4 | 3 | 3 | 2 | 5 | 3 | 5 | 4 | 4 | 4 | 7 | 3 |
| Ergonomics | - | - | - | - | - | - | - | - | - | 18 | - | - | 21 | - | - | 29 | 29 | 26 | - | - |
| Ethics | - | - | - | - | - | - | - | - | - | 17 | - | 16 | 21 | - | - | - | - | - | - | - |
| Ethnic Studies | - | - | - | - | - | - | - | - | - | - | - | - | - | - | - | 28 | - | - | - | - |
| Evolutionary Biology | 3 | - | - | 5 | - | 7 | 12 | - | 11 | - | 22 | 19 | 18 | 23 | 27 | 29 | 30 | 20 | 22 | 38 |
| Family Studies | - | - | - | - | - | - | - | - | - | 18 | - | - | - | - | 29 | - | - | - | 29 | - |
| Fisheries | - | - | 3 | - | - | - | 12 | 12 | - | 17 | - | 18 | - | 25 | 26 | 24 | 22 | 26 | 25 | 35 |
| Food Science & Technology | - | - | - | - | - | - | 13 | - | - | - | 20 | 18 | 21 | 24 | 25 | 28 | 31 | - | - | 27 |
| Forestry | 3 | - | 3 | 3 | 6 | 9 | 13 | 7 | 12 | 14 | 19 | 17 | 18 | 18 | 26 | 16 | 18 | 10 | 19 | 23 |
| Gastroenterology & Hepatology | - | - | - | - | - | - | - | - | - | - | - | - | - | 25 | 29 | - | 31 | - | 27 | - |
| Genetics & Heredity | - | - | - | - | - | - | - | - | 12 | - | 20 | 19 | 18 | - | 27 | 30 | 28 | 26 | 27 | 37 |
| Geochemistry & Geophysics | - | - | - | - | - | - | - | - | - | - | - | - | - | - | - | - | 31 | - | 28 | 39 |
| Geography | - | - | - | 5 | 7 | 4 | 11 | 11 | 10 | 15 | 9 | 9 | 16 | 16 | 20 | 21 | 20 | 20 | 27 | 34 |
| Geography Physical | - | - | - | 2 | - | 9 | 13 | 12 | 11 | 18 | 19 | 19 | 16 | 17 | 17 | 19 | 20 | 13 | 24 | 30 |
| Geosciences Multidisciplinary | 3 | - | - | - | - | 8 | 6 | 13 | 9 | 6 | 6 | 12 | 6 | 13 | 6 | 6 | 9 | 7 | 12 | 13 |
| Geriatrics & Gerontology | - | - | - | 5 | - | - | - | 9 | 11 | 12 | 20 | 18 | 16 | 18 | 16 | 18 | 24 | 17 | 20 | 26 |
| Gerontology | - | - | - | 5 | - | - | - | 9 | 11 | 12 | 21 | 18 | 16 | 18 | 18 | 19 | 25 | 19 | 22 | 28 |
| Green & Sustainable Science & Technology | - | - | 3 | 5 | - | - | - | - | 12 | - | 20 | 11 | 12 | 18 | 12 | 8 | 8 | 6 | 6 | 4 |
| Health Care Sciences & Services | - | - | - | - | - | - | - | - | - | 16 | 22 | - | 18 | 17 | 25 | 24 | 20 | 15 | 17 | 11 |
| Health Policy & Services | - | - | - | - | - | - | - | - | - | - | - | - | 20 | 24 | 28 | 25 | 23 | 21 | 28 | 18 |
| Hematology | - | - | - | - | - | - | - | - | - | - | - | 20 | - | 25 | - | - | - | - | 29 | 40 |
| History & Philosophy Of Science | - | - | - | - | - | - | - | - | 12 | - | - | 16 | 21 | - | - | 30 | 27 | - | - | - |
| Horticulture | - | - | - | - | - | - | - | - | - | - | - | - | - | - | 29 | - | - | - | - | - |
| Hospitality Leisure Sport & Tourism | - | - | - | - | - | - | - | - | - | - | - | - | - | 24 | - | - | 31 | - | - | 40 |
| Imaging Science & Photographic Technology | - | - | - | - | - | - | - | - | - | - | 22 | - | - | 24 | - | - | 30 | 25 | - | 39 |
| Immunology | - | - | - | 5 | - | - | - | - | - | 16 | - | 20 | 20 | 25 | 29 | - | - | 22 | 26 | 39 |
| Infectious Diseases | - | - | - | - | - | - | - | - | - | - | - | - | 20 | - | 29 | - | 30 | 26 | 22 | 39 |
| Information Science & Library Science | - | - | - | - | - | - | - | - | - | - | - | - | 20 | - | - | - | - | - | - | 40 |
| Instruments & Instrumentation | - | - | - | - | - | - | - | - | - | - | - | - | - | - | - | - | 31 | - | - | 39 |
| Integrative & Complementary Medicine | - | - | - | - | - | - | - | - | 12 | - | - | - | 20 | - | 28 | - | 30 | 26 | 26 | 38 |
| Limnology | - | - | - | - | - | - | - | 11 | 10 | 15 | 19 | 20 | - | 24 | 28 | 30 | 30 | 26 | 27 | 37 |
| Management | - | - | 3 | - | - | - | - | - | - | - | - | - | - | 23 | 25 | 27 | 29 | 25 | 22 | 31 |
| Marine & Freshwater Biology | - | - | 3 | 3 | 5 | 9 | 6 | 2 | 5 | 4 | 11 | 16 | 8 | 18 | 14 | 14 | 12 | 14 | 14 | 28 |
| Materials Science Characterization & Testing | - | - | - | - | - | - | - | - | - | - | - | - | - | - | - | - | 31 | - | - | - |
| Materials Science Multidisciplinary | - | - | - | - | - | - | - | - | - | - | - | - | - | - | - | - | 30 | - | - | 40 |
| Mathematical & Computational Biology | - | - | - | - | - | - | 13 | 13 | 12 | - | 20 | - | - | - | 27 | 29 | - | - | - | - |
| Mathematics | - | - | - | - | - | - | - | - | - | - | - | - | - | - | - | - | - | - | - | 40 |
| Mathematics Applied | - | - | - | - | - | - | - | - | - | 18 | - | - | - | - | - | - | - | - | - | - |
| Mathematics Interdisciplinary Applications | - | - | - | - | - | - | - | - | 12 | - | 17 | - | 21 | 18 | 28 | 30 | 30 | 24 | 29 | 37 |
| Medical Ethics | - | - | - | - | - | - | - | - | - | 18 | - | - | - | - | - | - | - | - | - | - |
| Medical Informatics | - | - | - | - | - | - | - | - | - | 18 | 22 | - | - | 24 | - | - | 31 | 25 | 26 | 40 |
| Medicine General & Internal | - | - | - | 5 | - | - | 13 | 12 | - | 18 | 22 | 20 | 21 | 23 | 25 | 28 | 30 | 17 | 20 | 21 |
| Medicine Legal | - | - | - | - | - | - | - | - | - | - | - | - | - | - | - | - | - | 23 | 29 | 40 |
| Medicine Research & Experimental | - | - | - | - | - | - | - | - | - | - | 22 | - | - | 23 | 29 | 27 | 29 | - | 28 | 32 |
| Meteorology & Atmospheric Sciences | - | - | - | - | - | - | 7 | - | 11 | 10 | 10 | 9 | 9 | 16 | 7 | 11 | 13 | 6 | 11 | 8 |
| Microbiology | - | - | - | - | - | - | 13 | - | - | - | 22 | 19 | 18 | 25 | 26 | 29 | 31 | 21 | 29 | 37 |
| Multidisciplinary Sciences | - | - | 3 | 5 | - | 6 | - | - | 12 | 17 | 21 | 20 | 20 | 20 | 20 | 23 | 24 | 20 | 28 | 35 |
| Mycology | - | - | - | - | - | - | - | - | - | - | - | - | - | - | - | - | - | 26 | - | - |
| Nanoscience & Nanotechnology | - | - | - | - | - | - | - | - | - | - | - | - | - | - | - | - | 31 | - | - | - |
| Neuroimaging | - | - | - | - | - | - | - | - | 12 | - | 21 | - | 20 | 25 | - | 29 | - | - | 27 | - |
| Neurosciences | - | - | - | 4 | - | 7 | 11 | 10 | 5 | 7 | 8 | 5 | 4 | 5 | 6 | 13 | 6 | 5 | 8 | 9 |
| Nursing | - | - | - | - | 7 | - | 10 | - | 9 | 8 | 14 | 8 | 11 | 11 | 12 | 7 | 3 | 8 | 4 | 6 |
| Nutrition & Dietetics | - | - | - | - | - | - | 13 | - | - | - | 21 | - | - | - | 29 | 28 | - | 25 | 29 | 38 |
| Obstetrics & Gynecology | - | - | - | - | - | - | - | - | 12 | - | 21 | 18 | 20 | 25 | - | 30 | 31 | 25 | 22 | 39 |
| Oceanography | - | - | 3 | 5 | 7 | - | 12 | 3 | 8 | 11 | 18 | 18 | 16 | 25 | 13 | 20 | 19 | 21 | 22 | 36 |
| Oncology | - | - | - | - | - | - | 11 | - | - | 12 | 21 | 19 | - | 12 | 24 | 24 | 22 | 16 | 16 | 24 |
| Operations Research & Management Science | - | - | 3 | - | - | - | - | - | - | - | 17 | 16 | 21 | 21 | 19 | 22 | 23 | 15 | 18 | 22 |
| Ophthalmology | - | - | - | - | - | - | - | - | - | - | - | - | - | - | - | - | - | - | 29 | - |
| Optics | - | - | - | - | 7 | 9 | - | 13 | - | 18 | 22 | - | - | 24 | - | - | - | - | - | - |
| Ornithology | - | - | - | - | - | - | - | 13 | - | - | - | - | - | - | - | - | - | - | - | - |
| Orthopedics | - | - | - | - | - | - | - | - | - | - | - | 16 | - | 25 | - | - | 31 | - | 25 | 35 |
| Otorhinolaryngology | - | - | - | - | - | - | - | - | - | - | - | - | - | - | 29 | - | - | - | - | 40 |
| Paleontology | - | - | - | - | - | - | - | - | - | - | - | - | - | - | - | - | - | 26 | 29 | - |
| Parasitology | - | - | - | 5 | - | 8 | - | 13 | 11 | 18 | 22 | - | 20 | - | 29 | - | 29 | 26 | 27 | 40 |
| Pathology | - | - | - | - | - | - | - | - | - | - | 22 | - | - | - | - | - | - | - | - | - |
| Pediatrics | - | - | - | 4 | - | 8 | 11 | - | 10 | 16 | 17 | 16 | 21 | 22 | 26 | 21 | 15 | 12 | 15 | 15 |
| Peripheral Vascular Disease | - | - | - | - | - | - | - | - | - | - | - | - | - | - | - | - | - | - | - | 40 |
| Pharmacology & Pharmacy | - | - | - | - | - | - | - | 12 | 11 | 13 | 21 | 10 | 6 | 21 | 25 | 27 | 21 | 11 | 23 | 21 |
| Physics Applied | - | - | - | - | - | - | - | - | - | - | - | - | - | - | - | - | - | - | - | 40 |
| Physics Multidisciplinary | - | - | - | - | - | - | - | - | - | - | - | - | - | - | - | - | 31 | - | - | - |
| Physiology | - | - | - | - | - | - | - | - | - | - | - | 19 | - | - | 29 | 30 | 31 | 26 | 29 | 39 |
| Plant Sciences | 3 | - | - | 3 | 3 | - | 13 | 13 | 12 | 15 | 17 | 16 | - | 16 | 26 | 17 | 18 | 12 | 13 | 30 |
| Primary Health Care | - | - | - | - | - | - | - | - | - | - | - | - | - | - | - | 30 | - | - | 28 | 40 |
| Psychiatry | - | - | - | 4 | 6 | 6 | 5 | 5 | 3 | 1 | 4 | 4 | 2 | 4 | 3 | 3 | 2 | 2 | 2 | 2 |
| Psychology | - | - | - | 4 | - | - | 7 | 10 | 8 | 9 | 16 | 10 | 10 | 14 | 15 | 14 | 14 | 10 | 10 | 12 |
| Psychology Applied | - | - | - | - | - | - | 12 | - | - | 17 | - | - | - | 21 | 29 | 27 | 27 | 24 | 29 | 39 |
| Psychology Biological | - | - | - | - | - | - | - | - | - | - | - | 19 | - | 25 | 15 | 30 | 27 | 24 | 29 | 37 |
| Psychology Clinical | - | - | - | - | - | - | - | 13 | 7 | 12 | 13 | 20 | 16 | 23 | 25 | 20 | 21 | 17 | 15 | 16 |
| Psychology Developmental | - | - | - | 5 | - | - | - | - | 12 | 14 | 19 | 20 | 17 | 23 | 24 | 27 | 25 | 22 | 23 | 30 |
| Psychology Educational | - | - | - | - | - | - | - | - | - | - | - | - | 21 | - | - | - | - | - | - | - |
| Psychology Experimental | - | - | - | - | - | - | - | - | - | - | - | 19 | - | - | 29 | 29 | 31 | 25 | - | 40 |
| Psychology Mathematical | - | - | - | - | - | - | - | - | - | - | - | - | 21 | - | - | - | - | - | - | - |
| Psychology Multidisciplinary | - | - | - | - | - | - | 9 | - | - | 18 | 21 | 16 | 21 | 20 | 23 | 29 | 30 | 20 | 21 | 36 |
| Public Environmental & Occupational Health | - | - | - | - | 7 | - | - | - | 9 | 9 | 7 | 6 | 8 | 6 | 10 | 15 | 10 | 7 | 5 | 5 |
| Radiology Nuclear Medicine & Medical Imaging | - | - | - | - | - | - | - | - | - | - | 21 | - | - | 25 | - | - | - | 26 | 27 | 40 |
| Regional & Urban Planning | - | - | - | 5 | - | - | - | 13 | - | - | - | - | - | 25 | 25 | 30 | 27 | 23 | 29 | 40 |
| Rehabilitation | - | - | - | - | - | - | - | - | 10 | 17 | - | 17 | 19 | 15 | 26 | 29 | 27 | 25 | 22 | 27 |
| Remote Sensing | - | - | - | - | - | - | - | - | - | - | - | - | - | 24 | - | - | 30 | 24 | - | 37 |
| Reproductive Biology | - | - | - | - | - | - | - | - | - | - | 21 | - | 20 | - | - | - | - | - | - | - |
| Respiratory System | - | - | - | - | - | - | 13 | - | - | - | - | 20 | - | - | - | 29 | 29 | 26 | 26 | 40 |
| Rheumatology | - | - | - | - | - | - | - | - | - | 18 | - | 18 | - | - | 29 | - | 30 | - | 28 | 35 |
| Robotics | - | - | - | - | - | - | - | - | - | - | - | - | 21 | - | - | - | - | - | - | - |
| Social Issues | - | - | - | - | - | - | - | - | - | 18 | - | - | - | - | - | - | - | - | - | - |
| Social Sciences Biomedical | - | - | - | - | - | - | 11 | - | - | 17 | 17 | 16 | 21 | 24 | 24 | 28 | 30 | 22 | 21 | 32 |
| Social Sciences Interdisciplinary | - | - | - | - | - | - | - | - | - | - | - | - | - | - | - | 30 | - | 26 | - | 39 |
| Social Sciences Mathematical Methods | - | - | - | - | - | - | - | - | 12 | - | 17 | - | - | 19 | 28 | 30 | - | 26 | 29 | 39 |
| Social Work | - | - | - | - | - | 9 | - | - | 11 | 10 | 21 | 19 | 21 | - | - | 30 | - | - | - | - |
| Sociology | - | - | - | - | - | - | - | - | 12 | - | - | - | - | - | - | 30 | 30 | - | - | - |
| Soil Science | 2 | - | 2 | 4 | 3 | 9 | 13 | 13 | - | 18 | 21 | 20 | 14 | - | 24 | 26 | - | 26 | - | 40 |
| Sport Sciences | - | - | - | - | - | - | - | - | - | - | - | - | - | 24 | - | 30 | 29 | 25 | 29 | 39 |
| Statistics & Probability | - | - | - | - | - | - | - | - | - | - | - | - | - | - | - | - | - | - | - | 39 |
| Substance Abuse | - | - | - | 4 | - | - | - | 11 | - | 17 | 17 | - | 19 | 24 | 28 | 26 | - | 24 | 28 | 38 |
| Surgery | - | - | - | 5 | - | - | - | - | 12 | - | - | 20 | 21 | 19 | 29 | 26 | 28 | 25 | 20 | 32 |
| Telecommunications | - | - | - | 5 | 5 | 9 | 11 | 13 | - | 18 | 19 | 19 | - | 24 | 28 | - | 30 | 25 | 27 | 20 |
| Thermodynamics | - | - | - | - | - | - | - | - | - | - | - | 20 | - | - | 29 | 30 | - | - | - | 40 |
| Toxicology | - | - | - | - | - | 9 | - | - | - | 17 | 22 | - | 21 | 23 | 29 | 30 | 30 | 24 | 28 | 40 |
| Transplantation | - | - | - | - | - | - | - | - | - | - | - | 20 | - | - | - | 29 | - | - | - | 38 |
| Transportation | - | - | - | - | - | - | - | - | - | - | - | 19 | - | - | 28 | 27 | 31 | 23 | 28 | 33 |
| Transportation Science & Technology | - | - | - | - | - | - | 13 | - | - | - | - | 19 | - | - | 26 | 27 | 31 | 23 | 25 | 30 |
| Tropical Medicine | - | - | - | - | - | - | - | - | - | - | - | 20 | 21 | - | - | - | 29 | - | 28 | - |
| Urban Studies | - | - | - | 5 | - | - | - | 13 | - | - | - | - | - | 23 | 25 | 27 | 27 | 19 | 29 | 39 |
| Urology & Nephrology | - | - | - | - | - | - | - | - | - | 18 | - | - | - | - | - | 30 | - | 23 | - | - |
| Veterinary Sciences | - | - | - | 5 | 7 | 7 | - | 13 | 11 | 18 | 22 | - | 21 | 21 | 28 | 26 | 25 | 26 | 27 | 32 |
| Virology | - | - | - | - | - | - | - | - | - | - | - | - | 21 | - | 29 | - | - | - | 26 | - |
| Water Resources | 3 | - | - | 5 | - | 9 | 4 | 12 | 9 | 5 | 5 | 7 | 8 | 7 | 4 | 5 | 7 | 6 | 9 | 7 |
| Womens Studies | - | - | - | - | - | - | - | - | - | - | - | - | - | - | - | - | - | - | 29 | - |
| Zoology | - | - | - | - | - | - | - | - | 12 | - | 21 | 20 | 20 | - | 27 | - | - | - | 28 | - |
